# Reversible photodissipation of composite photochromic azobenzene-alginate supramolecular hydrogels†

**DOI:** 10.1039/d1ra09218a

**Published:** 2022-02-09

**Authors:** Anna-Lena Leistner, David Georg Kistner, Christian Fengler, Zbigniew L. Pianowski

**Affiliations:** Institut für Organische Chemie Karlsruher Institut für Technologie Campus Süd, Fritz-Haber-Weg 6 76131 Karlsruhe Germany pianowski@kit.edu; Institut für Technische Chemie and Polymerchemie Karlsruher Institut für Technologie Campus Süd, Engesserstraße 18 76128 Karlsruhe Germany; Institute of Biological and Chemical Systems – FMS Karlsruher Institut für Technologie Campus Nord, Hermann-von-Helmholtz Platz 1, 76344 Eggenstein-Leopoldshafen Germany

## Abstract

Supramolecular smart materials can quickly elicit macroscopic changes upon external stimulation. Here we report that an azobenzene-containing cyclic dipeptide can form composite supramolecular hydrogels with alginate based on the charge complementarity, at lower loading than the critical gelation concentrations of either component. The gels can reversibly dissipate to fluids with UV light. They can also encapsulate and photorelease fluorescent cargo. Upon treatment of the gels with aqueous calcium salts, the alginate component is permanently cross-linked and the photochromic component is solubilized.

## Abstract 2

Supramolecular smart materials can quickly elicit macroscopic changes upon external stimulation with light.

## Graphical abstract

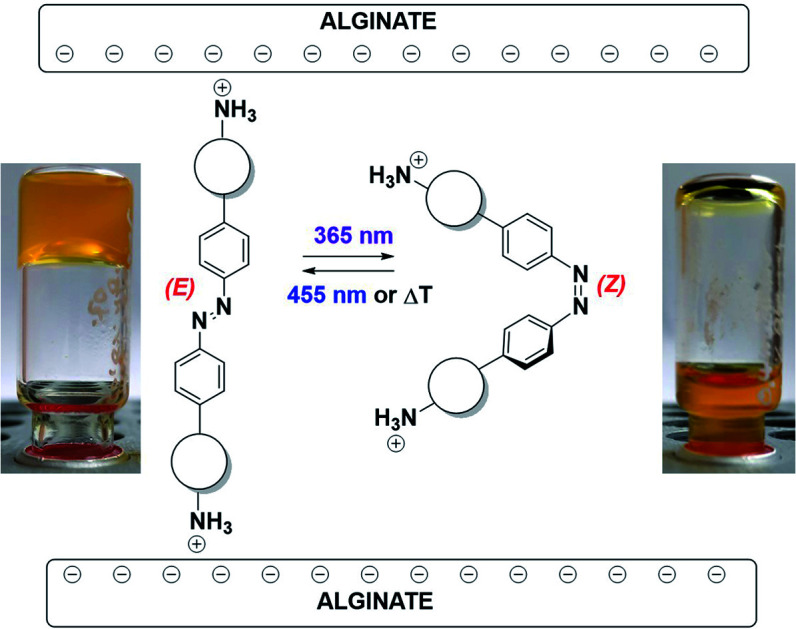

Photoresponsive supramolecular hydrogels are emerging smart materials^1,2^ with prospective applications in drug delivery^3–6^ or regenerative medicine.^7–9^ They are formed from hydrogelators, often based on sugars or oligopeptides, which self-assemble in water. Photoisomerization can disturb the self-assembly processes by changing the geometry or polarity of the photochromic component,^10^ and thus the way it interacts with other binding fragments or other photochromic molecules. In particular, our group has explored a series of low-MW supramolecular hydrogels based on the cyclic dipeptide (2,5-diketopiperazine, DKP) gelators,^11^ which reversibly disassemble to fluids upon exposure to UV^12^ or green light.^13,14^ These materials can efficiently release previously encapsulated antibiotics and anticancer drugs upon the light-driven dissipation. Upon stabilization, they might undergo microformulation to form injectable drug delivery vehicles. Therefore, we were interested in exploring the ways of increasing the mechanical stability or decreasing loading of the photochromic gelator, while maintaining the favorable stimuli responsiveness of the material. One possible strategy is to introduce further stabilizing supramolecular bonding,^15^ like electrostatic interactions,^16^ in addition to the π–π stacking and the hydrogen bonding network that stabilized the gelator fibers in earlier designs.^12–14^

Numerous composite soft materials based on supramolecular co-assembly of polysaccharides with short peptides have been demonstrated by the groups of Stupp,^17,18^ Ulijn,^19^ Adams,^20,21^ Mata^22–24^ or Williams,^25,26^ heading towards regenerative medicine applications. It was also shown, that photomodulation of composite photochromic hydrogels containing azobenzene as the light-triggered component binding with cyclodextrines installed on biocompatible polymers, like polyacrylates^27,28^ or alginate,^29–31^ can cause macroscopic changes of their physical properties. Thus, we wondered if a simpler system, based on electrostatic stabilization between the alginate carboxylic groups and a basic photochromic linker can be designed, which would maintain similar stimuli responsiveness.

In this communication, we report synthesis and photophysical characterization of the basic photochromic low-MW-hydrogelator 1 (Fig. 1), as well as properties of composite hydrogels prepared by mixing 1 with acidic low-MW alginate.

**Fig. 1:**
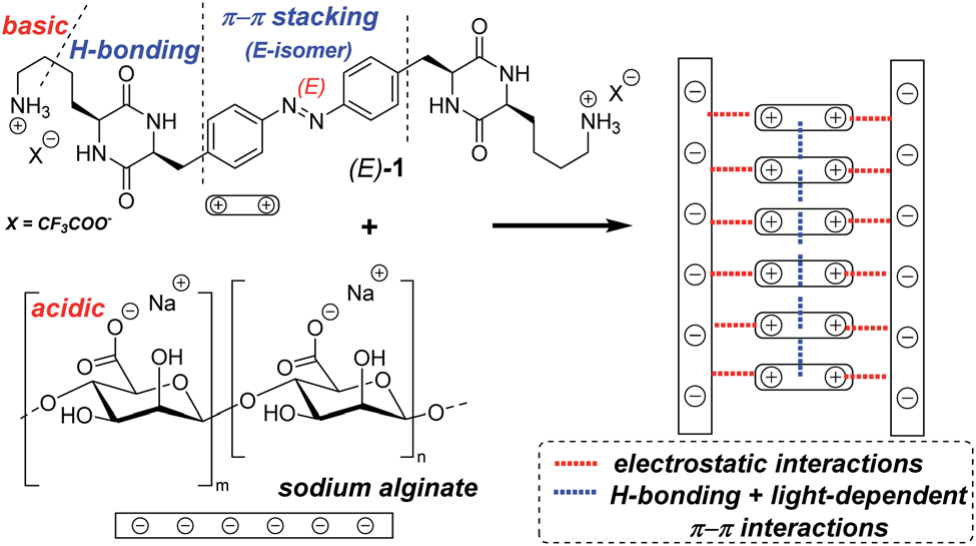
The bifunctional photochromic low-MW-hydrogelator 1 contains the azobenzene core with light-dependent polarity, hydrogen-bonding cyclic dipeptide (DKP) motifs, and alkylamine side chains that can produce stabilizing electrostatic interactions with acidic or poly-acidic additives. The right-hand-side scheme depicts hypothetical assembly of the components in fibres of our hydrogels.

The gelation of such mixtures occurs at concentrations, which are significantly below the critical gelation concentration of either component. The resulting gels demonstrate good thermal stability, as well as distinct morphology at the microscale (SEM/TEM) from hydrogels solely based on 1. Both compositions maintain the reversible light-induced gel dissipation ability, like the gelators discussed in our previous reports.^12–14,32^

The basic lysine side chains in our previous DKP-based materials^12–14,32^ can interact with oppositely charged components, as shown *e.g.* by reduced leaking of acidic drug molecules physically encapsulated in the hydrogels,^13^ as well as by the substantial enhancement of hydrogel's thermal stability observed upon addition of various amounts (10–20 wt% of the dry hydrogelator mass) of polyanionic DNA oligomers.^12^ Therefore, we hypothesized that a hydrogelator bearing two alkylamine side chains of lysine residues (known to be fully protonated under neutral pH) could act as a photochromic supramolecular cross-linker with polymers exposing multiple acidic groups. This could, for example, result in formation of hydrogels at the significantly lower material loading, than the one required for hydrogels based solely on photochromic DKPs.

The respective hydrogelator 1 has been synthesized starting from (*S*)-4-nitrophenylalanine 2 as depicted in Scheme 1.

**Scheme 1:**
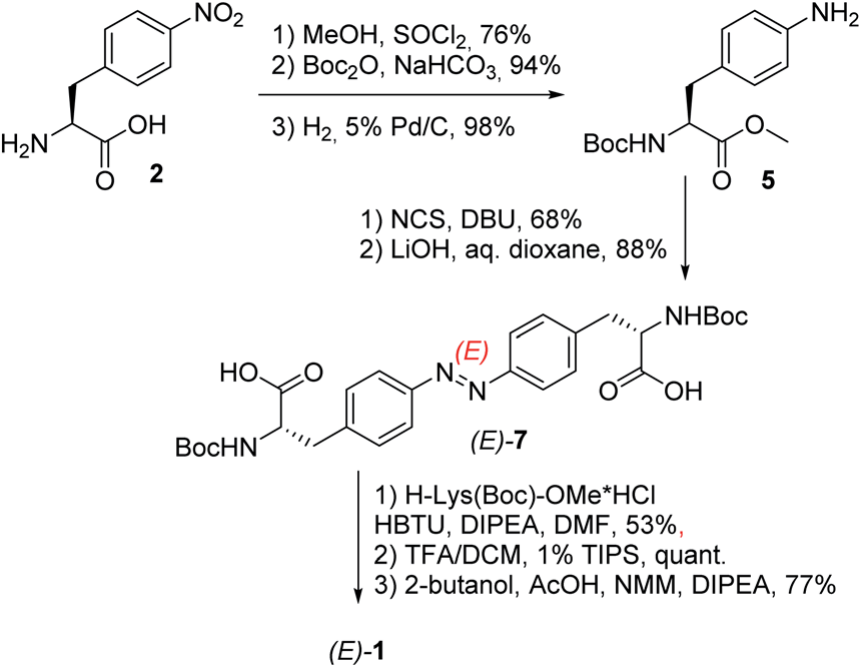
Synthesis of the low-MW-hydrogelator (*E*)-1.

Upon protection of its carboxylic and amino groups as a methyl ester and a Boc group, respectively, followed by reduction of the nitro group to the respective amine, we obtained a protected version of (*S*)-4-aminophenylalanine 5. This compound was oxidatively dimerized to the respective symmetric azobenzene (*E*)-6 bearing two amino acid substituents. Upon removal of the methyl esters (yielding (*E*)-7), the resulting carboxylic groups were coupled to a protected lysine. The linear bis-dipeptide 8 was then deprotected and cyclized to the expected bis-DKP-azobenzene hydrogelator 1.

Compound 1 was then characterized with regard to its photophysical and mechanical properties. Using ^1^H NMR spectroscopy, we determined the relative ratio of photoisomers at photostationary states (PSS) upon exposure to UV and visible light frequencies (PSS_365nm_ = 76% (*Z*)-1, PSS_455nm_ = 20% (*Z*)-1, Fig. S1 and S2†). Then, we measured thermal stability of the *Z*-isomer of 1 at room and elevated temperatures. The half-life of *Z*-1 was determined to be 9 days at 20 °C, or 158 min at 60 °C (both in diH_2_O) – the latter value comparable to an unsubstituted azobenzene at the same conditions.^33^ The thermal stability of (*Z*)-1 in acetic acid decreased more than 30-fold (Fig. 2 and S3–S5†).

**Fig. 2:**
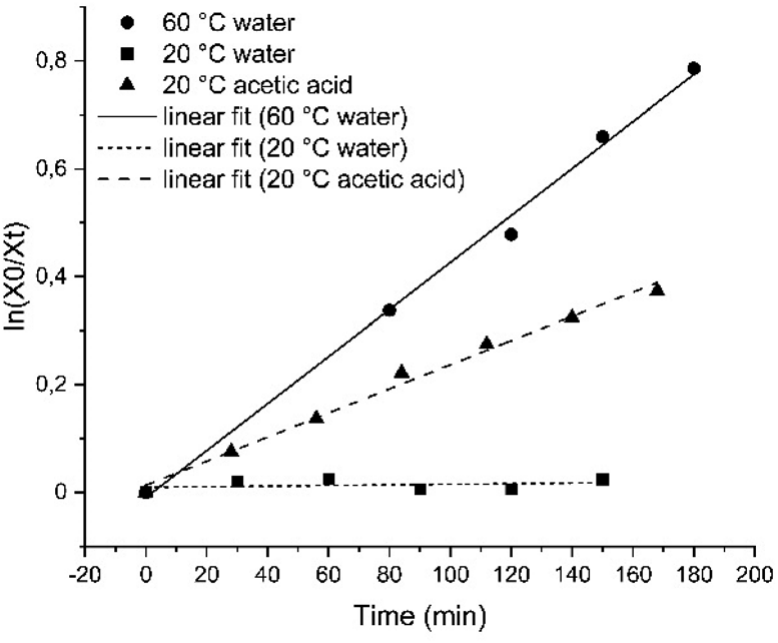
Linear fit of the decay of the *Z*-isomer of compound 1 for first-order kinetics in H_2_O at 20 °C (squares) or at 60 °C (circles), as well as in acetic acid at 20 °C (triangles).

Photochromism of 1 was determined with UV-vis spectroscopy for the thermally equilibrated *E*-1, as well as for the mixtures of photoisomers obtained upon photoequilibration of the *E*-1 with 365 nm (predominantly *Z*-1) and with 455 nm (predominantly *E*-1) light frequencies (Fig. 3).

**Fig. 3:**
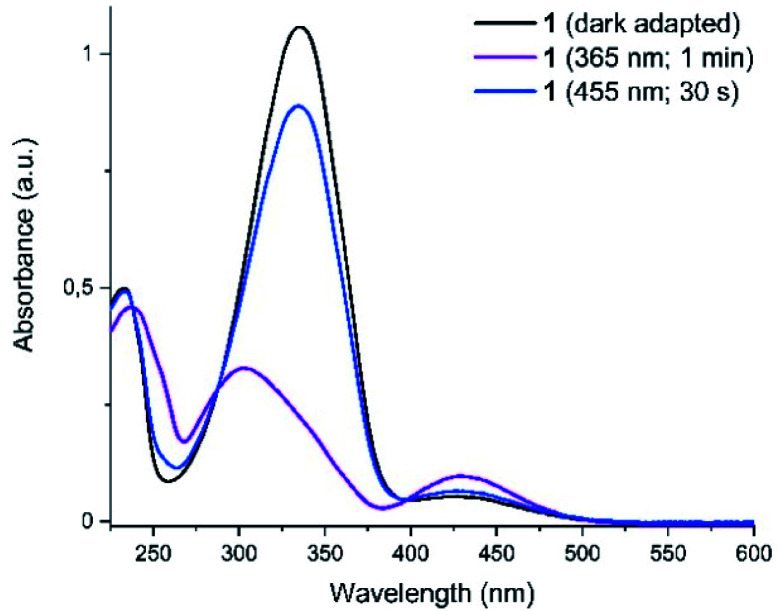
UV-vis spectra of the compound 1 in darkness, after irradiation with UV (365 nm) and with blue (455 nm) light. The curves after irradiation correspond to the photostationary states and distinct isosbestic points are displayed.

We have also determined the cytotoxicity of 1 against mammalian cells (HeLa) by MTT cell viability assays. For both formulations (the dark-stable *E*-isomer, and the 365 nm-photoequilibrated mixture containing majority of the *Z*-1), the cytotoxicity was low (IC_50_ at or above 1 mM, Table S5†).

Then we determined the propensity of 1 to form hydrogels. In neither water, 200 mM aq. NaCl, nor in Ringer's solution did compound 1 form hydrogels with any concentration tested. Yet, in PBS buffer (pH 7.4) the gelation occurred in the range between 1 wt% and 3 wt%, resulting in opaque orange gels, although the 1 wt% concentration resulted in unstable material sensitive to shaking (Table S1†). The hydrogel composed of 1.5 wt% of 1 in PBS (melting temperature – *T*_m_ = 77 °C) was selected for further experiments as gel A. This behavior indicates, that efficient hydrogelation of 1 may depend on the presence of multivalent anions capable of cross-linking and stabilizing the fibrous aggregates. Therefore, addition of a polymer that exposes multiple acidic groups on a single chain became an attractive perspective.

For that, we examined the ability of 1 to form composite supramolecular hydrogels with alginate – a polysaccharide (depicted on Fig. 1) with each subunit bearing a carboxylic group. In this case, the most optimal gelation has been observed by mixing components in deionized water. First, we tested the preparation technique (“method A”), where equivalent (1 : 1 by weight) mixtures of 1 and alginate were mixed, suspended in water, shortly boiled and then cooled to room temperature overnight in darkness (Table S2†). In the range of 0.6–1 wt% (the final concentration of each component), opaque orange gels with high melting temperatures were formed. The composition of 0.6 wt% of 1 + 0.6 wt% of alginate (*T*_m_ = 83 °C), has been investigated further as gel B.

Both, gel A and gel B, dissipated into fluids upon irradiation with UV light (365 nm, 15 min) (Fig. 4).

**Fig. 4:**
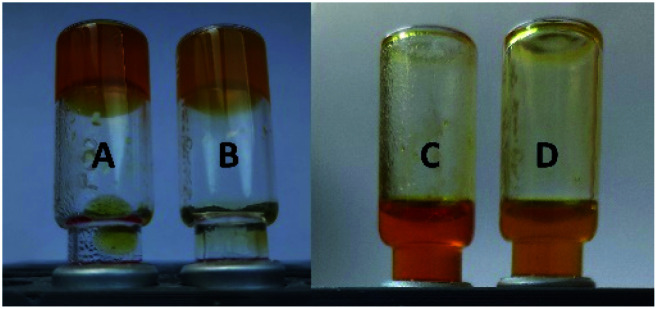
The gels composed of 1.5 wt% 1 in PBS buffer pH 7.4 (gel A, samples A and C), and the 0.6 wt% of 1 + 0.6 wt% of alginate (gel B, samples B and D) before (left) and after (right) UV light irradiation (365 nm for 15 min).

Subsequently, we tuned the composition by increasing the alginate-to-1 ratio. Stable hydrogels (*T*_m_ = 76–83 °C) have been generated at the 1 : 2–1 : 4 ratio (Fig. 5). Even the composition of 0.4 wt% of 1 + 0.8 wt% of alginate (gel C: *T*_m_ = 55 °C) remained mechanically stable.

**Fig. 5:**
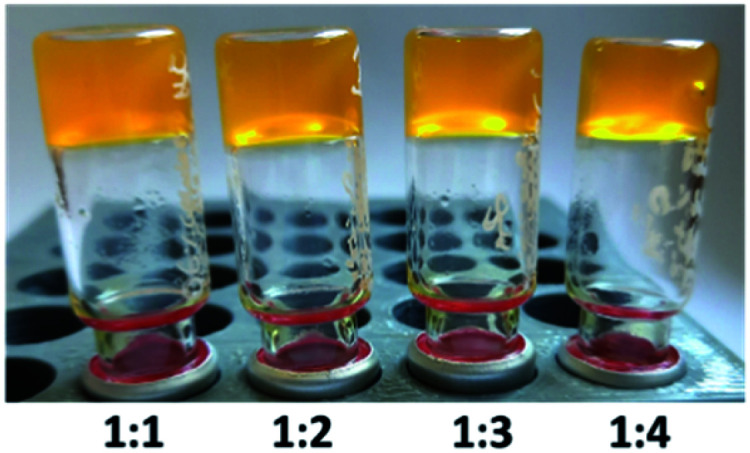
Gels composed of 0.6 wt% of 1 + alginate (0.6 wt%, 1.2 wt%, 1.8 wt%, and 2.4 wt%, respectively) in diH_2_O.

To examine reversible photodissipation of our materials, we have irradiated gel B (a hydrogel sample composed of 0.6 wt% of 1 + 0.6 wt% of alginate) with UV light (365 nm, 15 min). By that time, the sample was fully dissipated into fluid. Upon following irradiation with blue light (455 nm, 15 min), the fluid directly reconstituted a gel (Fig. S7†), while the same fluid incubated in darkness remained liquid for at least next three hours (it slowly solidifies in darkness due to thermal azobenzene back-isomerization to the *E*-isomer).

The morphology of gel A and gel B was investigated at the microscopic level with SEM (Fig. 6, S18 and S19†) and TEM (Fig. 7 and S14–S17†). Noteworthy, irradiation of gel A results in almost complete dissipation of the visible structures. Gel B retains a subtle fiber network, most likely composed of the alginate, upon liquefaction, while visibly losing the more complex threads that may originate from the superior cross-linking capacity of (*E*)-1*versus* its photoisomer (*Z*)-1.

**Fig. 6:**
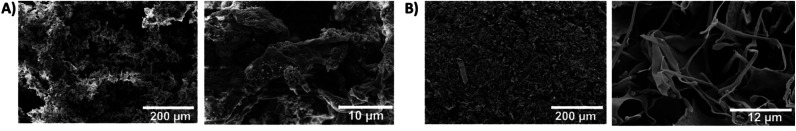
Scanning electron microscopy (SEM) of the hydrogels demonstrates morphological differences between the simple and the composite material. (A) gel A (1.5 wt% of 1 in PBS pH 7.4); and (B) gel B (0.6 wt% of 1 + 0.6 wt% of alginate in diH_2_O); both materials depicted at a magnification of 500× (left, scalebar 100 µm) and 10.000× (right, scalebar 10 µm for gel A and 6 µm for gel B).

**Fig. 7:**
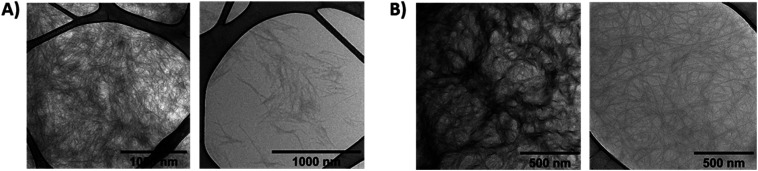
Transmission electron microscopy (TEM) of the hydrogels (without additional staining). (A) gel A (1.5 wt% of 1 in PBS pH 7.4) – dark-adapted (left) and irradiated with 365 nm UV light (right); (B) gel B (0.6 wt% of 1 + 0.6 wt% of alginate in diH_2_O) – dark-adapted (left) and irradiated with 365 nm UV light (right).

Next, we performed rheological investigations of a gel sample composed of 0.6 wt% 1 and 1.2 wt% of alginate. While both separate components (1.2 wt% alginate, and 0.6% 1 in diH_2_O) remained proper liquids, the viscoelastic behavior of the composite material could be quantified. Fig. 8 shows a frequency sweep at a constant strain amplitude *γ* = 0.5% within the linear viscoelastic regime (strain sweep – Fig. S20†).

**Fig. 8:**
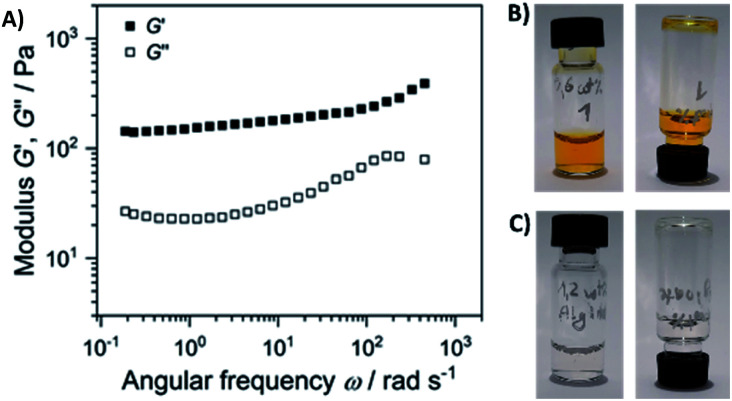
(A) Elastic *G*′ and loss modulus *G*″ as a function of angular frequency of the sample composed of 0.6 wt% 1 and 1.2 wt% of alginate (the gel depicted on Fig. 5). Frequency sweep was performed at a strain amplitude of 0.5% and 20 °C; the separate components are non-viscous fluids: (B) 0.6 wt% solution of 1, and (C) 1.2 wt% solution of alginate.

The elastic modulus *G*′ exceeds the loss modulus *G*″ by approximately one magnitude with tan *δ* = *G*″/*G*′ in the range of 0.30 to 0.15, indicating a predominant elastic response of the material. In addition, *G*′ is nearly independent of the frequency. Both factors suggest the formation of a physical gel formed through weak interactions of alginate and gelator 1, suggesting that 1 acts as a cross-linker that connects the linear polysaccharide chains.

Afterwards, we examined the influence of pH on the gelation process in the composite mixtures with the same component proportions as gel B, using a panel of buffers. The samples solidified in the range between pH 4 and pH 8 (Fig. 9 and Table S3†). At pH 10 no gelation was observed, but precipitation instead. This can be easily explained by partial deprotonation of the alkylamine side chain (p*K*_a_ = 10.53, pI = 9.74), which results in decreasing aqueous solubility and the ability to interact with alginate carboxylate groups.

**Fig. 9:**
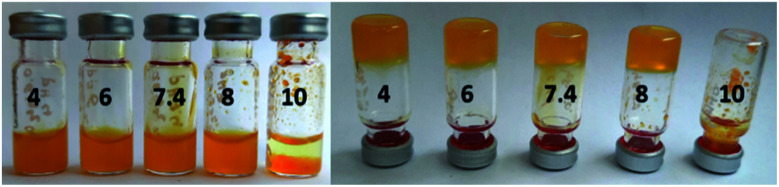
The hydrogels composed of 0.6 wt% 1 and 0.6 wt% of alginate in buffers. The respective pH values (in the range pH 4–pH 10) are indicated.

One potential application of our composite hydrogels is physical encapsulation and light-induced release of cargo compounds (*e.g.* for drug delivery). However, the initial hydrogel preparation method (method A) – with short boiling of the mixture components – could be detrimental for fragile guest substances. Thus, we wanted to develop a new gel loading protocol (further denoted as method B) compatible with heat-sensitive cargo (like many drugs, therapeutic antibodies, or even live cells). For that, the gelator 1 was initially dissolved in water and irradiated with UV light, to obtain the majority of *Z*-isomer. Then, it was mixed with solution of sodium alginate, eventually doped with a cargo substance, and the whole composition was irradiated with blue light, to cause the *Z* → *E* back-switching of 1. The gels were then incubated overnight in darkness. This preparation method resulted in slightly increased thermal stability of the resulting gels (Table S4†) (*T*_m_ of 89 °C *vs.* 83 °C for the composition of 0.6% 1 and 0.6% alginate prepared with method B and method A, respectively). And enabled for preparation of a stable transparent yellow gel with the lowest gelator input overall (0.3% 1 and 0.3% alginate, *T*_m_ = 80 °C) (Fig. 10 and S6†), used below for cargo photorelease.

**Fig. 10:**
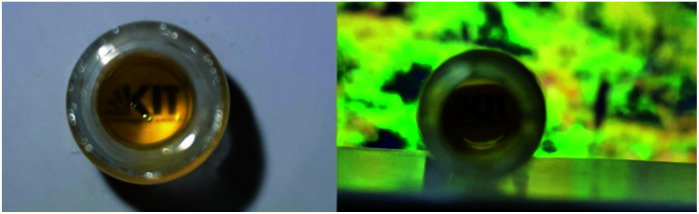
The transparent stable hydrogel composed of 0.3 wt% 1 and 0.3 wt% of alginate in water, prepared with method B.

To visualize the light-induced cargo release process, we decided to encapsulate fluorescent rhodamine B and compare the rate and efficiency of its photorelease with the spontaneous cargo leaking from our loaded gels in darkness. A sample of the gel loaded with cargo (500 µL of a gel composed of 0.3% 1 and 0.3% alginate, with 250 µg of rhodamine B) has been covered with equal volume of PBS buffer, which was replaced every 5 minutes with a fresh buffer aliquote. The amount of rhodamine B present in the supernatant was then quantified, and differed significantly between the sample irradiated with UV light, and the sample incubated in darkness. After 40 min (8 additional aliquots of buffer), the irradiated sample released in total 113 µg (>45%) of the fluorescent cargo, while the same sample in darkness released only 52 µg (<21%), mostly at the beginning of the experiment – through diffusion from the surface layers of the gel (Fig. 11, S8 and S9†).

**Fig. 11:**
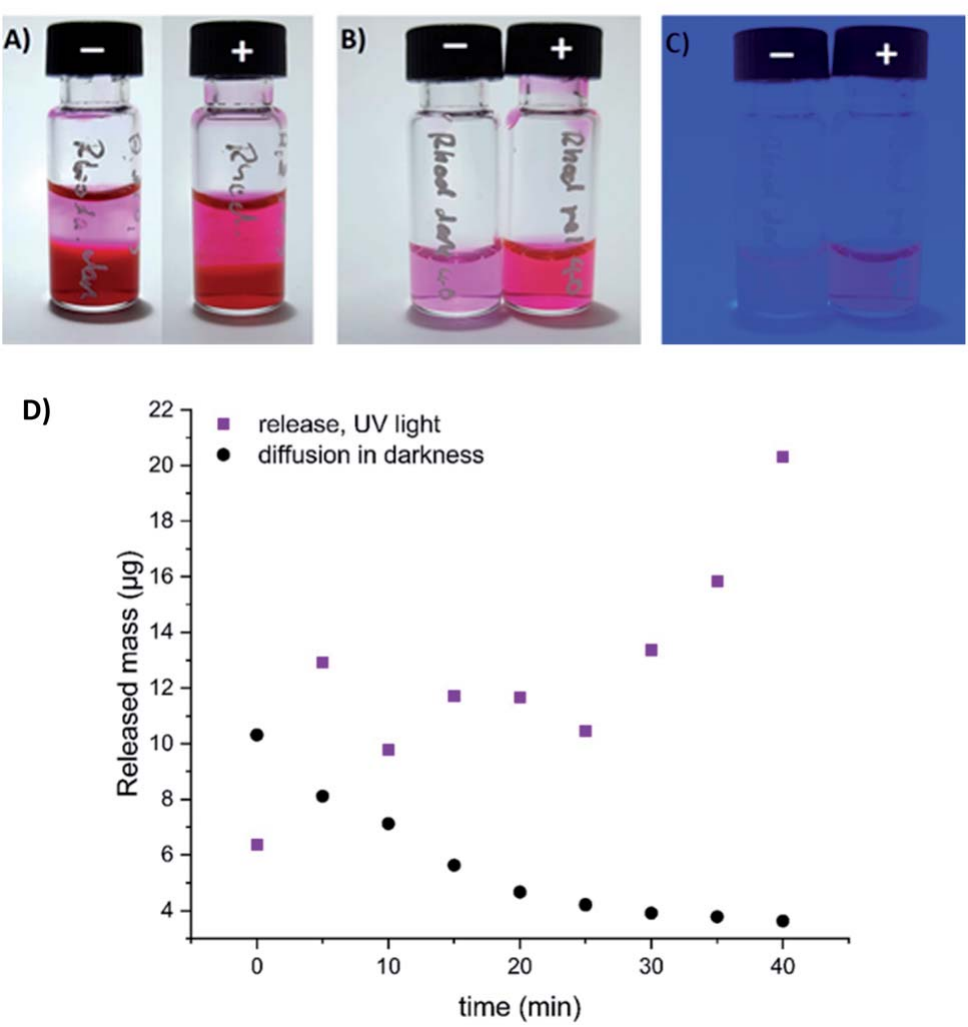
Spontaneous diffusion in darkness *vs.* light-induced release of fluorescent cargo (rhodamine B) from our composite hydrogel (0.3% of 1 and 0.3% alginate in water, method B). (A) gel loaded with rhodamine B, incubated with equivalent volume of PBS buffer in darkness (−), or upon irradiation with UV light (+) (the 9^th^ aliquot of buffer, 35–40 min. of the experiment); (B) the supernatant (9^th^ aliquot of buffer) collected over the rhodamine-loaded gel in darkness (−), or upon UV light irradiation (+) (under ambient light); (C) the same supernatant samples visualized under UV light irradiation; (D) quantification of released or diffused rhodamine B by HPLC (absorbance, converted to the released mass using a calibration curve depicted on Fig. S9†).

The rapid cargo release indicates utility of such composite materials as drug photorelease vehicles. The observed cargo leaking in darkness is still considerable, but for the DKP-based type of hydrogels it can be significantly reduced by tuning the cargo structure, as we have previously demonstrated.^14^

Finally, we also examined the effect of calcium salt solutions on our materials, because calcium ions are known to efficiently cross-link alginate and rapidly precipitate it from aqueous solutions. Two samples of a gel composed of 0.6 wt% of 1 and 0.6 wt% of alginate have been covered with equivalent volumes of 10% aqueous CaCl_2_ solution, which was exchanged for a fresh aliquot every hour, in total 4 times. One gel was irradiated with UV light, the other was incubated in darkness. In both cases, significant amounts of the yellow-colored gelator 1 have been observed in the supernatant (although this process was significantly more efficient for the irradiated sample). As the UV-light-irradiated sample has not been dissipated to fluid within 4 hours of duration of the experiment (in contrary to each previous sample of our composite gels irradiated in the absence of calcium ions), but instead remained a stable gel, we assume that the gelator 1 has been replaced to a significant extent with calcium ions, resulting in the non-photodissipative material (Fig. 12 and S11–S13†).

**Fig. 12:**
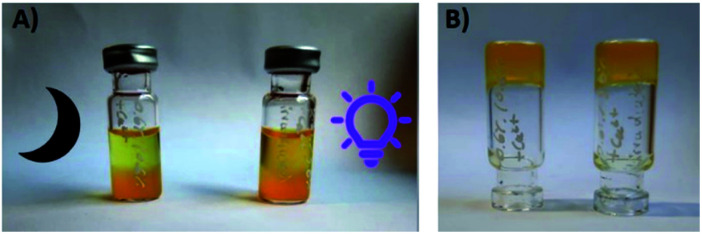
The composite hydrogel (0.6% of 1 and 0.6% alginate, method A) upon incubation with 10% aq. CaCl_2_ solution in darkness (left vial) or under UV light irradiation (right vial). (A) Significant amounts of 1 are observed in the supernatant in both cases, within 1 hour of incubation; (B) the remaining stable gel upon 4 hours incubation with 10% aq. CaCl_2_ solution.

As a consequence, we may anticipate that similar composite materials may be in the future 3D-photoprinted to a desired shape, and later permanently fixed in that shape upon further irradiation in a bath of aqueous calcium chloride solution. As the resulting calcium alginate is generally considered to be a biocompatible material, this may for example open up an avenue to encapsulation of stem cells inside our gels, and 3D-photoprinting of tailor-made implants, which – upon CaCl_2_ fixation and prolonged bathing in respective cell media – would gradually fill up with growing cells, producing *e.g.* bone fragments shaped for transplantations.

To conclude, we have demonstrated that an azobenzene, symmetrically substituted with two lysine-bearing DKP rings, efficiently forms composite supramolecular hydrogels with polyanionic alginate, significantly below the critical gelation concentration of 1 alone. The gels undergo complete dissipation to fluids upon exposure to UV light, and this process is reversible – thermally or upon exposure to blue light. The composite hydrogels are stable between pH 4 and pH 8. Electron microscopy analysis demonstrates distinct morphological differences caused by addition of the alginate, which correlate with the increased gelation propensity. Considering both EM and rheological analysis, we attribute gel formation in case of the composite materials to non-covalent interactions of 1 with alginate backbone (mainly electrostatic interactions of carboxylates and ammonium groups) where 1 acts as a cross-linker, and the alginate – as a stabilizing matrix. Hence, polysaccharides can be used as an additive to support structure formation of photochromic supramolecular hydrogels based on the DKP motif exposing basic side chains while maintain their light driven stimuli responsiveness.

Next, we have developed a method for loading our gels with heat-sensitive cargo. And we have demonstrated rapid light-triggered release of fluorescent cargo encapsulated in our material. Finally, upon treatment with aqueous CaCl_2_ solution, the composite hydrogel loses its photochromic component, and can be converted into a stable biocompatible soft material, permanently devoid of photosensitivity. The last observation can be useful for biocompatible 3D photoprinting. In the future, we want to optimize the structure of the photoswitch to enable visible-light-triggered hydrogel dissipation, important for printing materials containing *e.g.* living cells.

## Conflicts of interest

There are no conflicts to declare.

## Supplementary Material

RA-012-D1RA09218A-s001
