# Supplementary material for: Reversible photodissipation of composite photochromic azobenzene-alginate supramolecular hydrogels
Source: RSC Adv. 2022 Feb 9;12(8):4771–6. doi: 10.1039/d1ra09218a (PMC8981262; doi:10.1039/d1ra09218a)
Supplement: RA-012-D1RA09218A-s001 [file RA-012-D1RA09218A-s001.pdf]

Electronic Supplementary Material (ESI) for RSC Advances.
This journal is © The Royal Society of Chemistry 2022

Supplementary Information:

Reversible photodissipation of composite photochromic azobenzene-alginate supramolecular hydrogels

*Anna-Lena Leistner[a], David Georg Kistner[a], Christian Fengler[b] and Zbigniew L. Pianowski[a,c]**

[a] Institut für Organische Chemie, Karlsruher Institut für Technologie,
Fritz-Haber-Weg 6, 76131 Karlsruhe, Germany
[b] Institut für Technische Chemie und Polymerchemie, Karlsruher Institut für Technologie
Engesserstraße 18, 76128 Karlsruhe, Germany
[c] Institute of Biological and Chemical Systems – FMS, Karlsruher Institut für Technologie,
Hermann-von-Helmholtz Platz 1, Campus Nord, 76344 Eggenstein-Leopoldshafen, Germany
* pianowski@kit.edu

Table of contents:

1. General Information: 3
2. Synthesis: 5
3. Photophysical properties 12
4. Gelation experiments 16
5. Light induced gel-to-sol transition 21
6. Light-induced rhodamine release 22
7. Diffusion of the gelator 1 after addition of Ca^{2+} ions 25
8. Cell viability assays of the hydrogelator 1 28
9. Microscopy images 30
10. Rheological experiments 33
11. NMR spectra of the synthesized compounds 34
12. References 48

1. General Information:

All reagents and starting materials are commercially available (SIGMA-ALDRICH, FLUOROCHEM, CHEMPUR, ALFA AESAR or BEPHARM) and were used as supplied unless otherwise indicated. In particular, we have used low-MW alginate ("Alginic acid sodium salt, very low viscosity", Alfa Aesar cat.# A18565, viscosity: 7.0 mPa in 1% solution, certificate of analysis – **Figure S41**) to minimize entanglements of the polymer chains.All experiments were conducted in air and in deionized water (MILLIPORE) unless otherwise noted. All experiments with molecules that can photoisomerize were performed in absence of sunlight (brown glassware, or colorless glassware wrapped with aluminium foil, working in a room with dimmed light). All reactions containing air- and moisture-sensitive compounds were performed under argon using oven-dried glassware applying common Schlenk-techniques. Liquids were added via steel cannulas and solids were added directly in powdered shape.

Column chromatography was performed on Silica gel 60 Å (40-63 µm particle size) (Sigma). **NMR spectra** were recorded using the following device: ^{1}H NMR: Bruker Avance 400 (400 MHz), ^{13}C NMR: Avance 400 (101 MHz), ^{19}F NMR: Avance 400 (377 MHz). The following solvents from Eurisotop were used: chloroform-d_1 ($CDCl_3$), DMSO-d_6, and D_2O. Chemical shifts δ were expressed in parts per million (ppm) and referenced to $CDCl_3$ (^{1}H: δ=7.26 ppm, ^{13}C: δ=77.16 ppm), DMSO-d_6 (^{1}H: δ=2.50 ppm, ^{13}C: δ=39.52 ppm) and D_2O (^{1}H: δ=4.79 ppm). [1] ^{19}F-NMR were not referenced. **Mass spectra** were recorded on a Finnigan MAT 95 mass spectrometer using electron ionization-mass spectrometry (EI-MS) or fast atom bombardment-mass spectroscopy (FAB-MS). For FAB measurements *m*-nitrobenzyl alcohol (3-NBA) was used as the matrix. The software of FAB and EI adds the mass of one electron. Electrospray ionization–mass spectrometry (ESI-MS) spectra were recorded on a Thermo Fisher Scientific Q Exactive mass spectrometer. Calibration was carried out using premixed calibration solutions (Thermo Fisher Scientific). The molecular fragments are stated as ratio of mass per charge *m/z*. **UV-Vis spectra** were recorded on a Lambda 750 (PerkinElmer) UV-Vis spectrophotometer at 20 °C, slit=2 nm. Quartz cuvettes of 10 mm optical path length were used. **IR spectra** were recorded on a Bruker IFS 88 using ATR (Attenuated total reflection). The intensities of the absolute peaks are given as follows: vs=very strong 0-9% T, s=strong 10-39% T, m=medium 40-69% T, w=weak 70-89% T, vw=very weak 90-100% T. All spectroscopy samples were taken at room temperature. **Analytical High Performance Liquid Chromatography (HLPC)** was performed using a Thermofisher UltiMate 3000 system containing a degaser, pump, autosampler, column compartment and diode array detector. The flow rate was 1 mL/min on a stationary *PerfectSil Target* (MZ-Analytik) C_{18} column (3-5 µm, 4.0 mm × 250 mm). Chromeleon 7 software was used for data extraction. **Preparative HPLC** separation was performed with a LC-2000Plus series from Jasco with a VDSpher column with C18-M-SE, 250 × 20 mm and 10 µm from VDSoptilab.

Analytical thin layer chromatography was carried out using silica coated aluminium plates (silica 60, F_{254}, layer thickness: 0.25 mm) with fluorescence indicator by Merck. Detection proceeded under UV light at λ=254 nm.

Sample irradiation for measurements of photostationary states was performed using LED diodes with following emission maxima: 10 W LED diode: 365 nm from LED Engin and 3 W LED diode 455 nm from Avonec. For the time of irradiation, samples were maintained at constant temperature (22 ± 2 °C) using a metal cooling block unless otherwise noted.

Using the PowerMax USB (type PS19Q) sensor device (Coherent®) we have measured the irradiation intensity for the particular diodes used in our experiment (5 independent measurements, the detector (diameter 19 mm) was located at the distance of 55 mm from the light source, identical as the position of irradiated samples).

The optical power density for the light sources: 365 nm: 0.56 mW/cm^2 (SD 0.0308 mW/cm^2), 455 nm: 9.93 mW/cm^2 (SD 0.0003 mW/cm^2)

2. Synthesis:

L-Phe-(4-NO$_2$)-OH, (*S*)-4-Nitrophenylalanine (2)

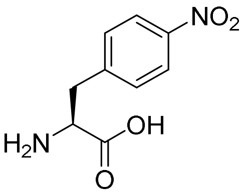

L-Phenylalanine (20.0 g, 121 mmol, 1.00 equiv.) was dissolved in 96% sulfuric acid (61 mL), heated up to 55 °C while vigorously stirring until everything dissolved, and then cooled down to 0 °C on an ice-water bath. When the internal temperature decreased below 5 °C, a mixture of 65% nitric acid (15.3 g, 16.9 mL, 243 mmol, 2.01 equiv.) and 96% sulfuric acid (24.4 g, 13.3 mL, 249 mmol, 2.06 equiv.) was added dropwise and the temperature was kept below 10 °C. After the addition was finished the reaction mixture was stirred for an additional 2.5 h and then poured on 200 mL of crushed ice. Using 25% aqueous ammonia solution the reaction mixture was neutralized to precipitate the crude product. After stirring overnight, the precipitate was filtered off and washed with ice-cold water (3×50 mL). The crude precipitate was recrystallized from water and dried under high vacuum yielding 11.8 g of a beige powder (56.2 mmol, 46%).

^{1}H NMR (400 MHz, D$_2$O with KOH): δ = 8.17 (d, J = 8.6 Hz, 1H), 7.44 (d, J = 8.6 Hz, 1H), 3.55 (t, J = 5.6 Hz, 1H), 3.07 (dd, J = 13.4, 5.9 Hz, 1H), 2.97 (dd, J = 13.4, 7.2 Hz, 1H) ppm. **^{13}C NMR (101 MHz, D$_2$O with KOH):** δ = 181.8, 146.8, 146.4, 130.2, 123.6, 57.3, 40.8 ppm. **TLC:** R_f = 0.45 (developed in 79% CH_2Cl_2, 20% MeOH, 1% Et_3N). **HRMS (EI+):** *m/z* calcd. for $C_9H_{10}N_2O_4$ [M] = 210.0635 Da, found 210.0637 Da (Δ = 0.7 ppm). **IR (ATR):** ṽ = 3291 (w), 3272 (w), 3259 (w), 3245 (w), 3234 (w), 3210 (w), 3200 (w), 3193 (w), 3109 (w), 3084 (w), 3065 (w), 3053 (w), 3043 (w), 2997 (w), 2982 (w), 2944 (w), 2901 (w), 2885 (w), 2877 (w), 2776 (w), 2752 (w), 2738 (w), 2725 (w), 2646 (w), 1697 (w), 1643 (w), 1611 (s), 1568 (s), 1534 (vs), 1514 (vs), 1494 (m), 1442 (m), 1417 (s), 1344 (vs), 1312 (s), 1293 (m), 1242 (w), 1207 (w), 1191 (w), 1176 (w), 1140 (w), 1105 (m), 1071 (m), 1013 (w), 946 (w), 877 (m), 863 (s), 844 (w), 815 (w), 768 (m), 744 (s), 717 (m), 697 (vs), 653 (s), 630 (m), 615 (m), 567 (w), 524 (vs), 492 (s), 459 (w), 443 (w), 416 (m), 395 (w), 378 (w) cm^{-1}.

L-Phe-(4-NO$_2$)-OMe · HCl, (*S*)-4-Nitrophenylalanine methyl ester hydrochloride (3)

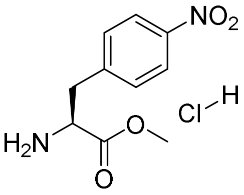

A suspension of 10.0 g L-Phe-(4-NO_2)-OH **2** (47.6 mmol, 1.00 equiv.) in 40 mL methanol was cooled to 0 °C on an ice-water bath. Then 25.9 mL thionyl dichloride (42.5 g, 357 mmol, 7.50 equiv.) were slowly added, the ice-water bath was removed, and the reaction mixture was stirred overnight at rt. The solvent was removed under reduced pressure and the residue dissolved again in 200 mL of methanol. The solution was added to vigorously stirred diethyl ether (200 mL) to precipitate the product, which was filtered off and washed with small amounts of diethyl ether. The product was dried under high vacuum yielding 9.39 g of a white powder (36.0 mmol, 76%).

^{1}H NMR (400 MHz, DMSO-*d*$_6$): δ = 8.81 (s, 3H), 8.23 – 8.15 (m, 2H), 7.62 – 7.53 (m, 2H), 4.37 (dd, *J* = 7.5, 6.0 Hz, 1H), 3.68 (s, 3H), 3.40 – 3.23 (m, 2H) ppm. **^{13}C NMR (101 MHz, DMSO-*d*$_6$):** δ = 169.0, 146.8, 143.1, 131.0, 123.6, 52.7, 52.7, 35.3 ppm. **TLC:** R_f = 0.36 (developed in 97% CH_2Cl_2, 3% MeOH). **HRMS (EI+):** *m/z* calcd. for $C_{10}H_{12}N_2O_4$ [M] = 224.0792 Da, found 224.0791 Da (Δ = − 0.4 ppm). **IR (ATR):** ṽ = 2982 (w), 2953 (w), 2907 (w), 2874 (w), 2847 (w), 1741 (vs), 1601 (w), 1541 (w), 1517 (s), 1506 (vs), 1490 (vs), 1451 (m), 1346 (vs), 1327 (m), 1309 (w), 1238 (vs), 1186 (m), 1146 (s), 1108 (m), 1060 (m), 980 (w), 949 (w), 932 (w), 868 (m), 858 (s), 844 (m), 812 (w), 751 (s), 741 (w), 700 (s), 654 (w), 507 (w), 490 (w), 405 (w) cm^{-1}.

Boc-L-Phe-(4-NO_2)-OMe, Methyl (*S*)-2-((*tert*-butoxycarbonyl)amino)-3-(4-nitrophenyl)-propanoate (4)

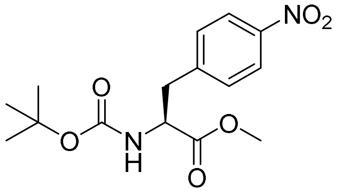

L-Phe-(4-NO_2)-OMe HCl **3** (8.50 g, 32.6 mmol, 1.00 equiv.) and 6.03 g of $NaHCO_3$ (71.7 mmol, 2.20 equiv.) were dissolved in 67 mL water. A solution of 7.83 g $(Boc)_2O$ (35.9 mmol, 1.10 equiv.) in 67 mL 1,4-dioxane was added dropwise. The reaction was stirred for 21 h at rt and stopped when reaction control via TLC showed full conversion. The solvent was removed under reduced pressure and the residue then redissolved in 100 mL water and 50 mL EtOAc. The mixture was extracted with EtOAc (3×30 mL). The combined organic layers were washed with 5% aqueous $KHSO_4$ solution (50 mL), 5% aqueous $NaHCO_3$ solution (20 mL), and brine (10 mL), then dried over Na_2SO_4. After filtration, the solvent of the filtrate was removed under reduced pressure. The product was dried under high vacuum yielding 9.89 g of a yellow powder (30.5 mmol, 94%).

^{1}H NMR (400 MHz, $CDCl_3$): δ = 8.18 – 8.13 (m, 2H), 7.34 – 7.28 (m, 2H), 5.05 (d, *J* = 7.8 Hz, 1H), 4.63 (q, *J* = 6.8 Hz, 1H), 3.73 (s, 3H), 3.27 (dd, *J* = 13.8, 5.8 Hz, 1H), 3.12 (dd, *J* = 13.7, 6.4 Hz, 1H), 1.41 (s, 9H) ppm. **^{13}C NMR (101 MHz, $CDCl_3$):** δ = 171.6, 154.9, 147.5, 144.0, 130.3, 123.7, 80.4, 54.1, 52.5, 38.4, 28.3 ppm. **TLC:** R_f = 0.65 (developed in 50% cH, 50% EtOAc). **HRMS (FAB+):** *m/z* calcd. for $C_{15}H_{20}N_2O_6$ [M+H] = 325.1394 Da, found 325.1395 Da (Δ = 0.2 ppm). **IR (ATR):** ṽ = 3356

(w), 2983 (w), 1728 (s), 1687 (s), 1676 (s), 1605 (w), 1598 (w), 1517 (vs), 1460 (w), 1451 (w), 1438 (w), 1415 (vw), 1391 (w), 1368 (w), 1343 (vs), 1320 (m), 1298 (s), 1269 (vs), 1251 (s), 1231 (s), 1193 (m), 1156 (vs), 1102 (m), 1057 (m), 1051 (m), 1033 (m), 1013 (m), 994 (m), 970 (w), 931 (w), 887 (w), 857 (s), 840 (s), 819 (w), 795 (w), 775 (w), 752 (s), 731 (w), 700 (m), 652 (m), 625 (m), 608 (m), 551 (w), 524 (w), 514 (w), 492 (w), 466 (w), 436 (w), 416 (w), 398 (w) cm^{-1}.

Boc-L-Phe-(4-NH_2)-OMe, Methyl (*S*)-3-(4-aminophenyl)-2-((tert-butoxycarbonyl)amino)-propanoate (5)

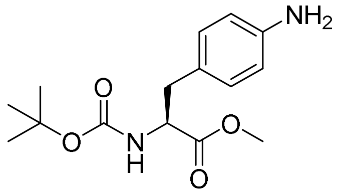

To a solution of 9.00 g Boc-L-Phe-(4-NO_2)-OMe **4** (27.7 mmol, 1.00 equiv.) in 50 mL MeOH was added 5% Pd/C (181 mg). The flask was set under vacuum and purged with Ar three times. After evacuating the flask one more time, hydrogen was added using a balloon. The reaction mixture was stirred vigorously and it was ensured that enough hydrogen was present. After 4 h and 24 h, additional 180 mg of 5% Pd/C were added. After 3 more hours, reaction control indicated full conversion and the reaction mixture was filtered through celite. The solvent was removed under reduced pressure and the product was dried under high vacuum yielding 8.00 g of a highly viscous, orange oil (27.2 mmol, 98%).

^{1}H NMR (400 MHz, $CDCl_3$): δ = 6.94 – 6.86 (m, 2H), 6.65 – 6.57 (m, 2H), 4.94 (d, *J* = 8.3 Hz, 1H), 4.50 (q, *J* = 6.6 Hz, 1H), 3.70 (s, 3H), 3.60 (br, 2H), 2.97 (q, *J* = 6.5, 4.7 Hz, 2H), 1.42 (s, 9H) ppm. **^{13}C NMR (101 MHz, $CDCl_3$):** δ = 172.7, 155.3, 145.5, 130.3, 125.8, 115.4, 80.0, 54.7, 52.3, 37.6, 28.5 ppm. **TLC:** R_f = 0.45 (developed in 50% cH, 50% EtOAc). **HRMS (FAB+):** *m/z* calcd. for $C_{15}H_{22}N_2O_4$ [M] = 294.1574 Da, found 294.1573 Da (Δ = − 0.4 ppm). **IR (ATR):** $\tilde{\nu}$ = 3437 (vw), 3424 (vw), 3411 (vw), 3366 (w), 3002 (vw), 2976 (w), 2952 (w), 2932 (w), 1737 (m), 1697 (vs), 1625 (m), 1517 (vs), 1500 (vs), 1438 (m), 1391 (m), 1366 (vs), 1278 (s), 1249 (s), 1215 (s), 1160 (vs), 1052 (s), 1016 (s), 992 (m), 922 (w), 856 (m), 824 (s), 802 (m), 778 (s), 759 (m), 728 (m), 654 (s), 637 (s), 626 (s), 608 (s), 565 (s), 534 (vs), 489 (s), 463 (s), 445 (s), 432 (s), 419 (s), 409 (s), 392 (s), 375 (s) cm^{-1}.

(*S*)-sym-$(Boc)_2$-PAP-OMe, Dimethyl 3,3'-(((*E*)-diazene-1,2-diyl)bis(4,1-phenylene))(2*S*,2'*S*)-bis(2-((tert-butoxycarbonyl)amino)propanoate) (6)

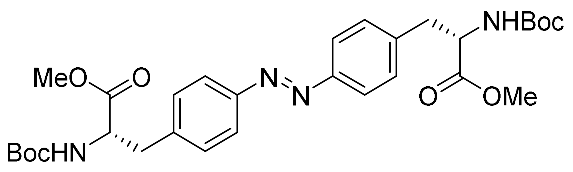

Boc-L-Phe-(4-NH_2)-OMe **5** (7.75 g, 26.4 mmol, 1.00 equiv.) was dissolved in 400 mL of dry CH_2Cl_2 and 7.85 mL DBU (8.01 g, 52.6 mmol, 2.00 equiv.) was added. The solution was stirred for 5 min at rt and then cooled down to −78 °C. NCS (7.03 g, 52.6 mmol, 2.00 equiv.) was added in small portions and the solution was stirred for 10 min. The mixture was quenched with 300 mL aqueous $NaHCO_3$ solution. The organic layer was separated, washed with water (100 mL) and 1M aqueous HCl (100 mL), and then dried over Na_2SO_4. The drying agent was filtered off and the solvent was removed under reduced pressure. Silica gel column chromatography was performed (73% cH, 27% EtOAc, to 70% cH, 30% EtOAc) to yield 5.01 g orange solid (8.99 mmol, 68%).

^{1}H NMR (400 MHz, DMSO): δ = 7.80 (d, J = 8.2 Hz, 4H), 7.45 (d, J = 8.1 Hz, 4H), 7.38 (d, J = 8.2 Hz, 2H), 4.25 (ddd, J = 10.0, 8.1, 5.2 Hz, 2H), 3.63 (s, 6H), 3.10 (dd, J = 13.8, 5.1 Hz, 2H), 2.96 (dd, J = 13.8, 10.1 Hz, 2H), 1.32 (s, 18H) ppm. **^{13}C NMR (101 MHz, DMSO):** δ = 172.4, 155.4, 150.7, 141.5, 130.2, 122.4, 78.3, 54.9, 51.9, 36.3, 28.1 ppm. **TLC:** R_f = 0.1 (developed in 73% cH, 27% EtOAc). **HRMS (FAB+):** m/z calcd. for $C_{30}H_{40}N_4O_8$ [M] = 584.2841 Da, found 584.2840 Da (Δ = − 0.2 ppm). **IR (ATR):** ṽ = 3376 (w), 2982 (w), 2953 (w), 1759 (m), 1740 (s), 1691 (vs), 1680 (vs), 1604 (vw), 1514 (vs), 1458 (w), 1436 (m), 1422 (w), 1391 (w), 1368 (m), 1353 (w), 1329 (w), 1293 (s), 1248 (vs), 1218 (s), 1162 (vs), 1147 (vs), 1105 (m), 1057 (m), 1037 (m), 1024 (s), 1010 (s), 989 (m), 956 (w), 922 (w), 892 (w), 868 (w), 847 (m), 782 (w), 759 (w), 722 (w), 698 (w), 684 (w), 677 (w), 667 (w), 656 (w), 642 (w), 586 (m), 561 (vs), 527 (m), 492 (w), 479 (w), 466 (w), 439 (w), 404 (w), 399 (w) cm^{-1}. **UV-Vis (MeCN):** λ_{max} = 232 nm, 331 nm.

(*S*)-sym-(Boc)$_2$-PAP-OH, (2*S*,2'*S*)-3,3'-(((*E*)-diazene-1,2-diyl)bis(4,1-phenylene))bis(2-((tert-butoxycarbonyl)amino)propanoic acid) (7)

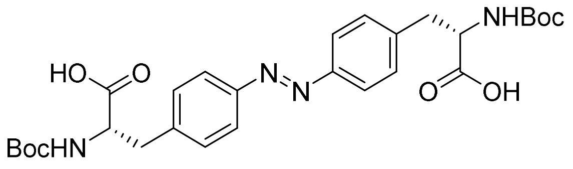

(*S*)-sym-(Boc)$_2$-PAP-OMe **6** (4.00 g, 6.84 mmol, 1.00 equiv.) was dissolved in 100 mL 1,4-dioxane and a solution of 9.83 g LiOH (409 mmol, 60 equiv.) in 100 mL water was added. After stirring for 15 min, 2M aqueous HCl (225 mL) was added and the mixture was extracted with EtOAc (2×250 mL). The combined organic layers were washed with brine (2×250 mL) and dried over anhydrous Na_2SO_4. After filtering off the drying agent, the solvent was removed under reduced pressure and the crude product was dried under vacuum. Silica gel column chromatography was performed to purify the product starting with 1% FA in CH_2Cl_2, then gradually MeOH was added

(1%, 1.5%, 2%, 3%) while keeping 1% FA in CH_2Cl_2. The product was obtained as orange solid (3.37 g, 6.05 mmol, 88%).

^{1}H NMR (400 MHz, DMSO): δ = 7.80 (d, J = 8.1 Hz, 4H), 7.46 (d, J = 8.1 Hz, 4H), 7.17 (d, J = 8.4 Hz, 2H), 4.22 – 4.11 (m, 2H), 3.13 (dd, J = 13.8, 4.6 Hz, 2H), 2.93 (dd, J = 13.9, 10.4 Hz, 2H), 1.32 (s, 18H) ppm. **^{13}C NMR (101 MHz, DMSO):** δ = 173.4, 155.4, 150.7, 142.0, 130.2, 122.3, 78.1, 54.9, 36.3, 28.1, 27.8 ppm. **TLC:** R_f = 0.19 (developed in 1% FA, 1% MeOH, 98% CH_2Cl_2). **HRMS (FAB+):** *m/z* calcd. for $C_{28}H_{36}N_4O_8$ [M] = 556.2528 Da, found 556.2529 Da (Δ = 0.2 ppm). **IR (ATR):** ṽ = 3361 (w), 2983 (w), 2938 (w), 2931 (w), 1713 (s), 1686 (vs), 1604 (w), 1517 (vs), 1446 (w), 1421 (w), 1391 (m), 1367 (m), 1324 (m), 1305 (m), 1293 (m), 1249 (s), 1235 (s), 1154 (vs), 1105 (w), 1051 (m), 1026 (w), 1014 (m), 986 (w), 936 (m), 895 (w), 857 (s), 836 (s), 816 (w), 778 (m), 748 (m), 667 (w), 643 (m), 623 (m), 603 (s), 569 (vs), 526 (m), 504 (w), 496 (w), 473 (w), 466 (w), 435 (m), 422 (w), 411 (w), 404 (w), 390 (m), 384 (m) cm^{-1}. **UV-Vis (MeCN):** λ_{max} = 234 nm, 331 nm.

Sym-(Boc)Lys$_2$-(Boc)PAP-OMe, Dimethyl 2,2'-(((2*S*,2'*S*)-3,3'-(((*E*)-diazene-1,2-diyl)bis(4,1-phenylene))bis(2-((tert-butoxycarbonyl)amino)propanoyl))bis(azanediyl))(2*S*,2'*S*)-bis(6-((tert-butoxycarbonyl)amino)hexanoate) (8)

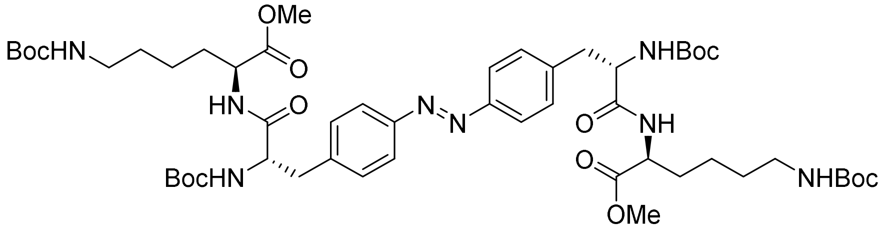

To a solution of 3.00 g (*S*)-sym-(Boc)$_2$-PAP-OH **7** (5.39 mmol, 1.00 equiv.) in anhydrous DMF (19 mL) was added 4.09 g HBTU (10.8 mmol, 2.00 equiv.) and 2.29 mL DIPEA (1.74 g, 13.5 mmol, 2.50 equiv.). The mixture was stirred for 10 min at rt under an Ar atmosphere before 3.22 g Methyl (2*S*)-2-amino-6-(tert-butoxycarbonylamino)hexanoate hydrochloride (H-L-Lys(Boc)-OMe·HCl, 10.8 mmol, 2.01 equiv.) and 2.29 mL DIPEA (1.74 g, 13.5 mmol, 2.50 equiv.) were added. After stirring for 2.5 h under an Ar atmosphere, the reaction was quenched by addition of 400 mL aqueous NH_4Cl solution which caused precipitation of an orange solid. The reaction mixture was extracted with 500 mL of EtOAc and the organic layer was washed with saturated aqueous NH_4Cl solution (2×400 mL) and with brine (400 mL). The organic layer was dried over Na_2SO_4, filtered and the solvent was removed under reduced pressure. The crude product was purified by silica gel column chromatography (R_f = 0.23 in 3% MeOH, 97% CH_2Cl_2, starting with pure CH_2Cl_2, then gradually addition of 0.5%, 1%, 2%, and 3% MeOH). The product **8** was obtained as orange solid (2.97 g, 2.85 mmol, 53%).

^{1}H NMR (400 MHz, DMSO-*d$_6$*): δ = 8.31 (d, J = 7.5 Hz, 2H), 7.80 (d, J = 8.0 Hz, 4H), 7.49 (d, J = 8.1 Hz, 4H), 6.97 (d, J = 8.7 Hz, 2H), 6.77 (d, J = 6.0 Hz, 2H), 4.27 (qd, J = 9.3, 8.5, 4.7 Hz, 4H), 3.62 (s, 6H), 3.11 – 2.77 (m, 8H), 1.67 (dt, J = 34.1, 7.6 Hz, 4H), 1.36 (s, 18H), 1.29 (s, 18H), 1.47 – 1.10 (m, 8H) ppm. **^{13}C NMR (101 MHz, DMSO-*d$_6$*):** δ = 172.5, 171.7, 155.6, 155.2, 150.6,

142.0, 130.3, 122.2, 78.1, 77.3, 55.3, 51.9, 51.8, 38.2, 37.3, 30.7, 29.1, 28.3, 28.1, 22.6 ppm. **TLC:** R_f = 0.23 (developed in 3% MeOH, 97% CH_2Cl_2). **HRMS (EI+):** *m/z* calcd. for $C_{52}H_{80}N_8O_{14}$ [M+H] = 1041.5867 Da, found 1041.5851 Da (Δ = − 1.5 ppm). **IR (ATR):** ṽ = 3333 (w), 3327 (w), 2976 (w), 2931 (w), 2864 (vw), 1742 (m), 1683 (vs), 1655 (vs), 1514 (vs), 1455 (m), 1443 (m), 1391 (m), 1366 (s), 1330 (w), 1306 (m), 1289 (m), 1268 (s), 1248 (vs), 1211 (s), 1163 (vs), 1132 (s), 1106 (m), 1045 (m), 1014 (s), 864 (w), 844 (m), 809 (w), 778 (w), 758 (w), 734 (w), 708 (w), 642 (s), 635 (m), 623 (m), 619 (m), 575 (s), 558 (m), 527 (m), 507 (w), 493 (w), 486 (w), 475 (w), 463 (w), 455 (w), 436 (m), 397 (w), 380 (w) cm^{-1}. **UV-Vis (MeCN):** λ_{max} = 234 nm, 331 nm.

Sym-PAP-Lys-OMe·TFA$_n$, Dimethyl 2,2'-(((2*S*,2'*S*)-3,3'-(((*E*)-diazene-1,2-diyl)bis(4,1-phenylene))bis(2-aminopropanoyl))bis(azanediyl))(2*S*,2'*S*)-bis(6-aminohexanoate) bis-(trifluoroacetate) (9)

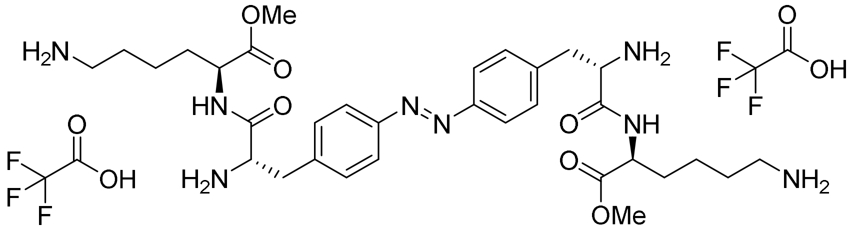

To a solution of 2.41 g sym-(Boc)Lys$_2$-(Boc)PAP-OMe **8** (2.31 mmol, 1.00 equiv.) in 110 mL CH_2Cl_2 was added 110 mL TFA and 1.10 mL TIPS. The mixture was stirred at rt for 1 h before 100 mL of toluene were added. Approximately 80% of the solvent was removed under reduced pressure. Another 100 mL of toluene were added and the solvent was evaporated under reduced pressure. After drying under high vacuum, the product was obtained as an orange TFA salt (3.35 g, 2.31 mmol, 100%).

^{1}H NMR (400 MHz, DMSO-*d$_6$*): δ = 8.98 (d, *J* = 7.5 Hz, 2H), 8.46 – 8.20 (m, 6H), 7.86 (s, 6H), 7.84 (d, *J* = 8.3 Hz, 4H), 7.49 (d, *J* = 8.2 Hz, 4H), 4.31 (td, *J* = 8.3, 5.5 Hz, 2H), 4.16 (d, *J* = 7.6 Hz, 2H), 3.62 (s, 6H), 3.15 (ddd, *J* = 54.8, 14.1, 6.7 Hz, 4H), 2.77 (dp, *J* = 11.6, 5.8 Hz, 4H), 1.82 – 1.21 (m, 12H) ppm. **^{13}C NMR (101 MHz, DMSO-*d$_6$*):** δ = 171.7, 168.1, 158.4 (q, *J* = 33.7 Hz), 151.2, 138.6, 130.7, 122.7, 116.5 (q, *J* = 295.8 Hz), 53.1, 52.1, 51.9, 38.5, 36.7, 30.5, 26.6, 22.1 ppm. **HRMS (FAB+):** *m/z* calcd. for $C_{32}H_{48}N_8O_6$ [M+H] = 641.3770 Da, found 641.3771 Da (Δ = 0.3 ppm). **IR (ATR):** ṽ = 3162 (w), 3132 (w), 3099 (w), 3078 (w), 3067 (w), 3058 (w), 3050 (w), 3040 (w), 3024 (w), 3013 (w), 2989 (w), 2955 (w), 2945 (w), 2927 (w), 2910 (w), 2894 (w), 2885 (w), 2871 (w), 2816 (w), 1731 (w), 1664 (vs), 1560 (m), 1550 (m), 1545 (m), 1527 (m), 1500 (m), 1475 (w), 1438 (m), 1431 (m), 1424 (m), 1357 (w), 1307 (w), 1295 (w), 1252 (w), 1177 (vs), 1126 (vs), 1013 (m), 997 (m), 984 (m), 907 (w), 888 (w), 836 (s), 798 (vs), 742 (w), 721 (vs), 705 (s), 660 (m), 643 (w), 598 (m), 581 (m), 562 (m), 552 (m), 517 (m), 493 (w), 480 (m), 472 (m), 465 (m), 458 (m), 433 (m), 414 (m), 399 (m), 388 (m) cm^{-1}. **UV-Vis (H_2O):** λ_{max} = 335 nm, 426 nm. **UV-Vis (MeCN):** λ_{max} = 327 nm.

PAP-DKP-Lys$_2$, (3*S*,3'*S*,6*S*,6'*S*)-6,6'-((((*E*)-diazene-1,2-diyl)bis(4,1-phenylene))bis(methylene))-bis(3-(4-aminobutyl)piperazine-2,5-dione) bis(trifluoroacetate) (1)

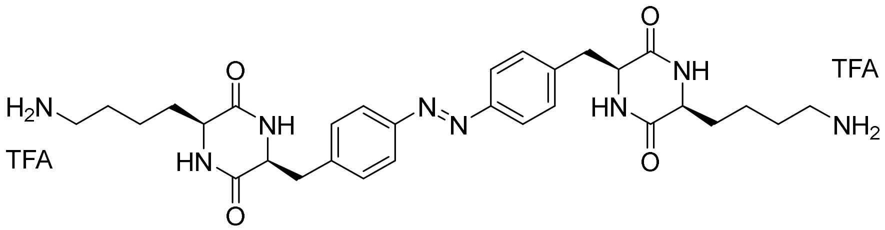

To a solution of the crude Sym-PAP-Lys-OMe·TFA$_n$ **9** (3.14 g, 2.16 mmol, 1.00 equiv.) in 192 mL 2-butanol was added 966 µL glacial AcOH (1.01 g, 16.9 mmol, 7.82 equiv.), 631 µL *N*-methylmorpholine (575 mg, 5.68 mmol, 2.63 equiv.), and 1.13 mL DIPEA (860 mg, 6.65 mmol, 3.08 equiv.). The mixture was heated to reflux (120 °C) and stirred for 2 h while orange solid precipitated. The mixture was cooled down and then concentrated by removing approx. half of the solvent under reduced pressure. After cooling down to rt, the solid was filtered off and washed with small amounts of ice-cold 2-butanol. The residue was dried under high vacuum to yield 1.33 g of an orange powder (1.65 mmol, 77%). For analytical purposes purification was done by preparative HPLC with the following settings: 15 mL/min, 11 min gradient 20-30% MeCN in bidest. H_2O with 0.1% TFA, detection at 330 nm, retention at 9.5 min. After lyophilization, the pure PAP-(DKP-Lys)$_2$ **(1)** was obtained as a TFA salt.

^{1}H NMR (400 MHz, DMSO-*d$_6$*): δ = 8.22 (d, *J* = 2.1 Hz, 2H), 8.12 (d, *J* = 2.2 Hz, 2H), 7.81 – 7.76 (m, 4H), 7.71 (s, 6H), 7.42 – 7.36 (m, 4H), 4.27 (td, *J* = 4.7, 2.3 Hz, 2H), 3.69 (t, *J* = 5.9 Hz, 2H), 3.21 (dd, *J* = 13.6, 4.4 Hz, 2H), 3.01 (dd, *J* = 13.6, 5.2 Hz, 2H), 2.65 – 2.53 (m, 4H), 1.36 – 1.19 (m, 6H), 1.06 – 0.86 (m, 6H) ppm. **^{13}C NMR (101 MHz, DMSO-*d$_6$*):** δ = 167.2, 166.5, 158.2 (q, *J* = 30.6 Hz), 151.0, 140.3, 131.3, 122.2, 55.2, 53.4, 38.5, 38.0, 32.4, 26.4, 20.6 ppm. **HRMS (FAB+):** *m/z* calcd. for $C_{30}H_{40}N_8O_4$ [M+H] = 577.3245 Da, found 577.3244 Da (Δ = – 0.3 ppm). **IR (ATR):** ṽ = 3187 (w), 3180 (w), 3167 (w), 3148 (w), 3140 (w), 3089 (w), 3075 (w), 3048 (w), 3004 (w), 2961 (w), 2929 (w), 2894 (w), 1664 (vs), 1561 (w), 1543 (w), 1534 (w), 1523 (w), 1499 (w), 1459 (m), 1432 (m), 1334 (m), 1303 (w), 1200 (s), 1180 (s), 1130 (vs), 1016 (w), 915 (w), 834 (s), 799 (s), 772 (m), 721 (s), 694 (w), 639 (w), 630 (w), 612 (w), 601 (w), 575 (w), 550 (w), 527 (w), 518 (w), 473 (m), 459 (w), 438 (s), 432 (s), 418 (m), 387 (w), 380 (w) cm^{-1}. **UV-Vis (H_2O):** λ_{max} = 335 nm, 426 nm.

3. Photophysical properties

Photostationary states determined by ^{1}H NMR measurements

Photostationary states were determined by ^{1}H NMR measurements for compound **1** (16.7 mg/mL) equilibrated up to 30 min under the indicated light wavelength (λ_{max} of the respective LED light diode). For analysis the NMR signals were assigned to the *E* and *Z* isomer, respectively. To determine the signals of the *Z* isomer the dark spectrum was subtracted from the spectrum after 30 min irradiation at 365 nm (10 W LED).

(*E*)-PAP-DKP-Lys$_2$ (1): ^{1}H NMR (400 MHz, DMSO-*d6*): δ = 8.22 (d, *J* = 2.1 Hz, 2H), 8.12 (d, *J* = 2.2 Hz, 2H), 7.81 – 7.76 (m, 4H), 7.71 (br s, 6H), 7.42 – 7.36 (m, 4H), 4.27 (tt, *J* = 4.9, 1.7 Hz, 2H), 3.69 (d, *J* = 5.9 Hz, 2H), 3.11 (ddd, *J* = 80.0, 13.6, 4.8 Hz, 4H), 2.58 (q, *J* = 6.7 Hz, 4H), 1.35 – 1.20 (m, 6H), 0.96 (tdt, *J* = 17.8, 7.9, 3.8 Hz, 6H) ppm.

(*Z*)-PAP-DKP-Lys$_2$ (1): ^{1}H NMR (400 MHz, DMSO-*d6*): δ = 8.17 (d, *J* = 2.0 Hz, 2H), 8.11 (d, *J* = 2.1 Hz, 2H), 7.78 (br s, 6H), 7.12 – 7.05 (m, 4H), 6.76 – 6.71 (m, 4H), 4.18 (td, *J* = 4.6, 2.2 Hz, 2H), 3.68 (d, *J* = 5.9 Hz, 2H), 2.94 (ddd, *J* = 97.2, 13.6, 4.6 Hz, 4H), 2.69 (tq, *J* = 11.3, 5.7 Hz, 4H), 1.39 (p, *J* = 7.2 Hz, 4H), 1.34 – 1.21 (m, 2H), 1.10 – 0.90 (m, 6H) ppm.

Then three signals per isomer were identified that did not—or just barely—overlapped with other signals. Those were integrated for each spectrum using the same intervals and divided by the number of protons. Each *E* isomer signal was assigned a *Z* isomer signal and the mole fraction of these pairs was calculated.

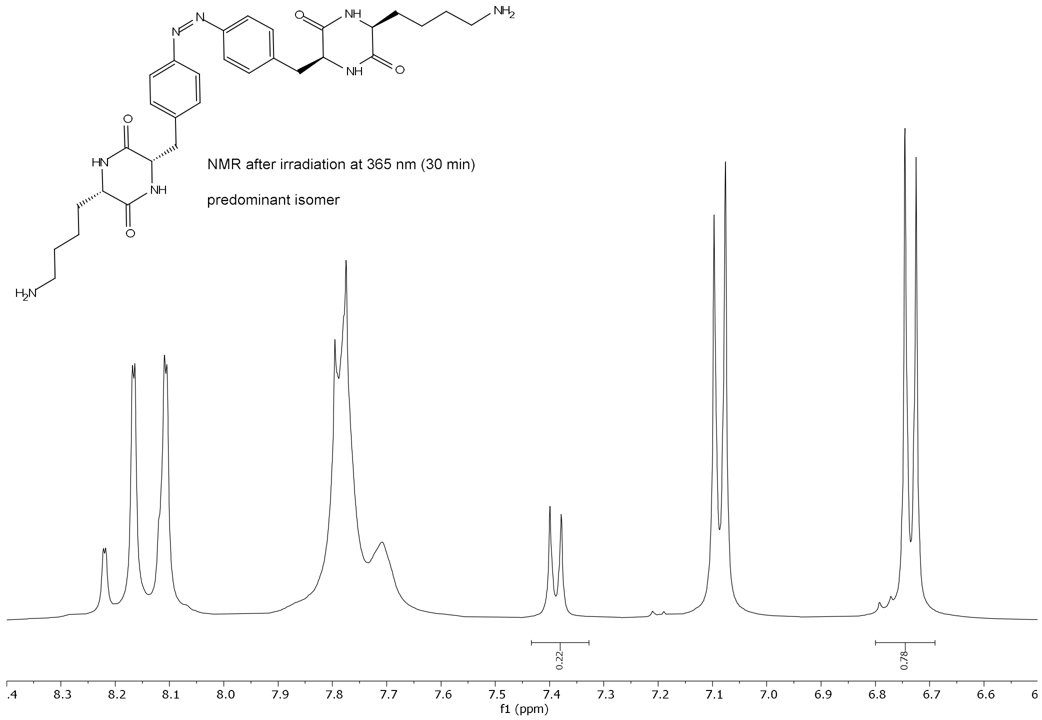

*Figure S 1: ^{1}H-NMR-spectrum (400 MHz, DMSO-d_6) of the compound **1** after irradiation at 365 nm. A photostationary state of **76% Z-isomer** is reached after 30 min irradiation time.*

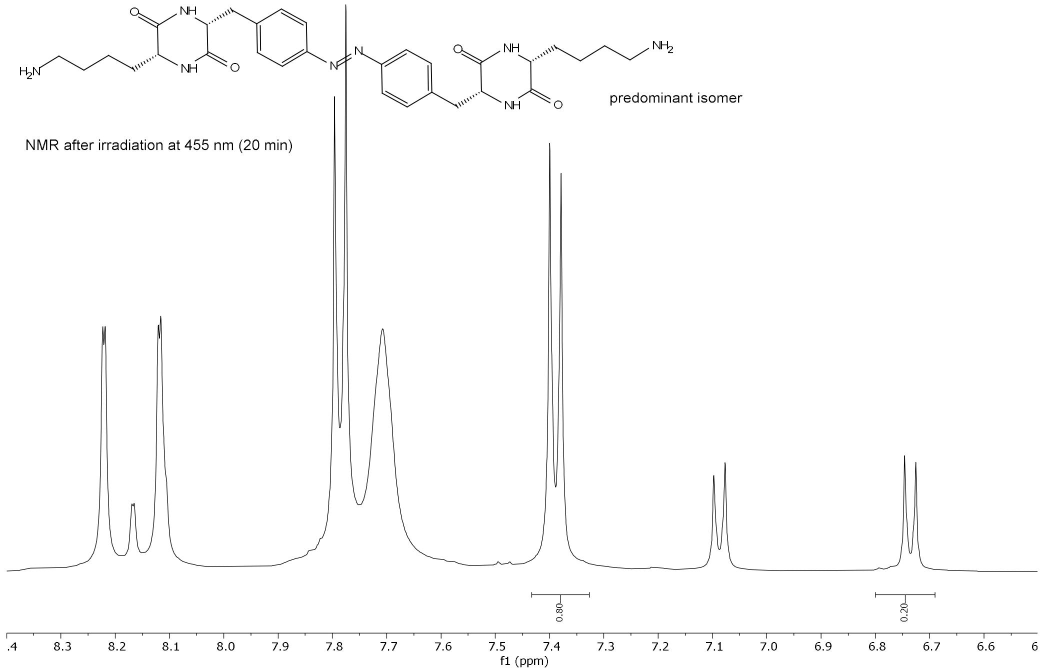

*Figure S 2: ^{1}H-NMR-spectrum (400 MHz, DMSO-d_6) of the compound **1** after irradiation at 455 nm. A photostationary state of **20% Z-isomer** is reached after 20 min irradiation time.*

Thermal stability

The thermal stability was determined for compound **1.** Samples were prepared in H_2O and acetic acid and irradiated at 365 nm to yield a high PSS. The solution was kept at 20 °C (H_2O and acetic acid) or at 60 °C (H_2O) and the isomer ratio was determined by HPLC in intervals. The obtained data was processed by calculating the ln(X_0/X_t), where X is the percentage of the respective *Z*-isomer and linear fitting (Equation (1)) of the obtained values. The calculated slope corresponds to the degradation rate constant k which is used to calculate the half-life $t_{1/2}$.

(1) $$x_t = x_0 \cdot e^{-k \cdot t} \leftrightarrow ln\left(\frac{x_0}{x_t}\right) = k \cdot t$$

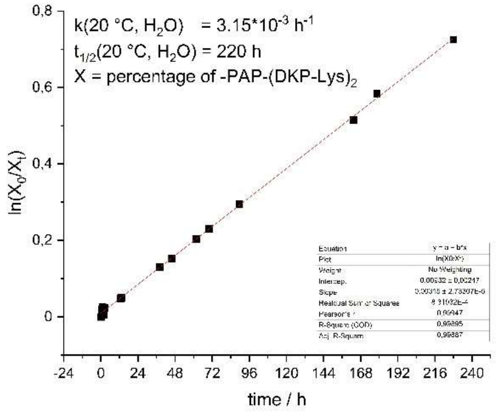

*Figure S 3: Linear fit of the decay of the Z-isomer of compound **1** at 20 °C in H_2O for first-order kinetics.*

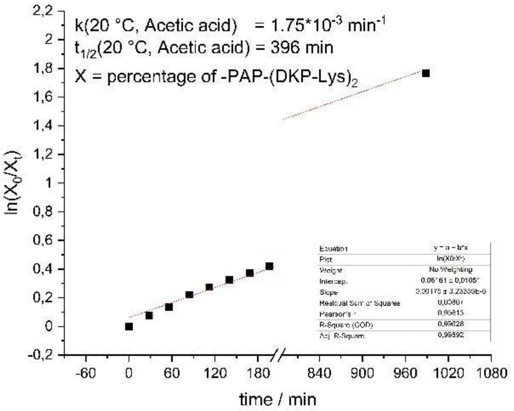

*Figure S 4: Linear fit of the decay of the Z-isomer of compound **1** at 20 °C in acetic acid for first-order kinetics.*

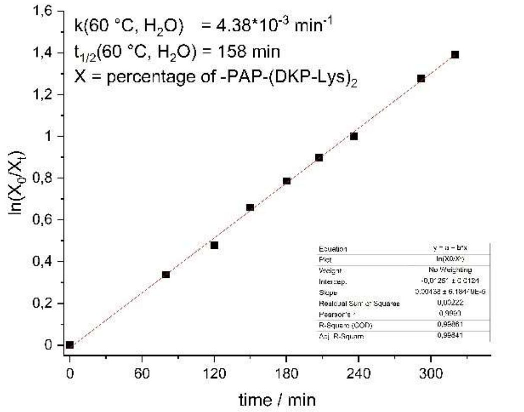

*Figure S 5: Linear fit of the decay of the Z-isomer of compound **1** at 60 °C in H_2O for first-order kinetics*

UV/Vis isomerization experiments

A 500 µM Stock solution was prepared and diluted to reach a final concentration of 50 µM. The cuvette with the sample was irradiated with light of different wavelengths (365 nm, 455 nm) directly before the measurement (see **Figure 3**).

Isosbestic points are determined at 238, 287 and 397 nm. Only the isosbestic point at 287 nm was used for quantification at HPLC.

4. Gelation experiments

Method A

To a 1.5 mL-vial (crimp top, 12×32 mm) was added the photochromic material **1** (and the additives) as powder and 500 µL of the aqueous solution. This suspension was treated by ultrasonic waves for 2 min followed by heating to 80 °C in a vial block. After equilibration at 80 °C for 5 min, the sample was heated to the boiling point by a heat gun. The hot solution became completely clear and upon cooling the hydrogels formed. Successfully formed gels were prepared in triplicates.

Melting temperatures were determined in triplicates. Gels were prepared as previously described. The vials were then mounted upside-down in a slowly stirred water bath (60 rpm) on a magnetic hotplate stirrer equipped with a thermometer. The water bath was heated (1.5 °C/min) until the gel started to melt and formed a sol.

Table S 1: Hydrogelation experiments without additives.

Composition of the solution (x mg of **1** + 500 µL water)	Approx. Concentration	Description	T_m °C
10	2.0 wt%	viscous orange liquid	-
2	0.4 wt%	viscous yellow liquid	-
1.5	0.3 wt%	viscous yellow liquid	-

Composition of the solution (x mg of **1** + 500 µL Ringer's solution)	Approx. Concentration	Description	T_m °C
10	2.0 wt%	viscous orange liquid	-
2	0.4 wt%	viscous yellow liquid	-
1.5	0.3 wt%	viscous yellow liquid	-

Composition of the solution (x mg of **1** + 500 µL 200 mM aq. NaCl)	Approx. Concentration	Description	T_m °C
10	2.0 wt%	viscous orange liquid	-
2	0.4 wt%	viscous yellow liquid	-

1.5	0.3 wt%	viscous yellow liquid	-

Composition of the solution (x mg of **1** + 500 µL PBS)	Approx. Concentration	Description	T_m °C
15	3.0 wt%	opaque, orange gel	-
10	2.0 wt%	opaque, orange gel	78
**7.5**	**1.5 wt%**	**opaque, orange gel**	**77**
5	1.0 wt%	Unstable orange gel, sensitive to shaking	69
2	0.4 wt%	viscous yellow liquid	-
1.5	0.3 wt%	viscous yellow liquid	-

Table S 2: Hydrogelation experiments with alginate – ***method A****.*

Composition of the solution (x mg + 500 µL H_2O)		Approx. Concentration		Description	T_m °C
Gelator **1**	Alginate	Gelator **1**	Alginate		
5	5	1.0 wt%	1.0 wt%	opaque, orange gel	-
4	8	0.8%	1.6%	opaque, orange gel	-
4	4	0.8 wt%	0.8 wt%	opaque, orange gel	-
3	12	0.6 wt%	2.4 wt%	opaque, orange gel	-
3	9	0.6 wt%	1.8 wt%	almost clear, orange gel	79
3	6	0.6 wt%	1.2 wt%	almost clear, orange gel	76
**3**	**3**	**0.6 wt%**	**0.6 wt%**	**opaque, orange gel**	**83**
3	2	0.6 wt%	0.4 wt%	unstable orange gel, sensitive to shaking	-
3	1.5	0.6 wt%	0.3 wt%	unstable orange gel, sensitive to shaking	-
2	6	0.4 wt%	1.2 wt%	clear yellow gel	57
2	4	0.4 wt%	0.8 wt%	clear yellow gel	55
2	2	0.4 wt%	0.4 wt%	viscous yellow liquid	-
1.5	6	0.3 wt%	1.2 wt%	unstable yellow gel, sensitive to shaking	-
0	5	-	1.0 wt%	viscous colorless liquid	-
0	4	-	0.8 wt%	colorless liquid	-
0	3	-	0.6 wt%	colorless liquid	-

*Table S 3: : Hydrogelation experiments at different pH (**method A**). **

Composition of the solution (x mg + 500 µL Buffer)		Approx. Concentration		Description	pH*
Gelator **1**	Alginate	Gelator **1**	Alginate		
3	3	0.6 wt%	0.6 wt%	opaque, orange gel, not stable upon shaking	4
3	3	0.6 wt%	0.6 wt%	opaque, orange gel, not stable upon shaking	6
3	6	0.6 wt%	1.2 wt%	opaque, orange gel, not stable upon shaking	6
3	3	0.6 wt%	0.6 wt%	opaque, orange gel, not stable upon shaking, precipitate	7.4
3	6	0.6 wt%	1.2 wt%	opaque, orange gel, not stable upon shaking	7.4
3	3	0.6 wt%	0.6 wt%	opaque, orange gel	8
3	3	0.6 wt%	0.6 wt%	Slightly colored liquid, precipitate	10
0	3	-	0.6 wt%	colorless liquid	-

*Buffer preparation:

pH=4

Components	Molecular weight (g/mol)	Concentration (mg/L)	mM
Citric acid	192	856	4.46
Potassium Chloride (KCl)	75.0	200	2.67
Sodium Chloride (NaCl)	58.0	8000	138
Sodium Phosphate dibasic (Na_2HPO_4-$7H_2O$)	268	292	1.09

The final exact pH was adjusted to 3.96 by addition of diluted aqueous NaOH solution and measuring the pH on a pH meter (WTW pH 3310 with a SenTix® 41 electrode).

pH=6

Components	Molecular weight (g/mol)	Concentration (mg/L)	mM
Potassium Chloride (KCl)	75.0	200	2.67
Sodium Chloride (NaCl)	58.0	8000	138
Sodium Phosphate monobasic (NaH_2PO_4)	120	1051	8.76
Sodium Phosphate dibasic (Na_2HPO_4-$7H_2O$)	268	330	1.23

The final exact pH was adjusted to 5.96 by addition of diluted aqueous HCl solution and measuring the pH on a pH meter (WTW pH 3310 with a SenTix® 41 electrode).

pH=8

Components	Molecular weight (g/mol)	Concentration (mg/L)	mM
Potassium Chloride (KCl)	75.0	200	2.67
Sodium Chloride (NaCl)	58.0	8000	138
Sodium Phosphate monobasic (NaH_2PO_4)	120	468	3.90
Sodium Phosphate dibasic (Na_2HPO_4-$7H_2O$)	268	1635	6.10

The final exact pH was adjusted to 8.02 by addition of diluted aqueous HCl solution and measuring the pH on a pH meter (WTW pH 3310 with a SenTix® 41 electrode).

pH=10

Components	Molecular weight (g/mol)	Concentration (mg/L)	mM

Potassium Chloride (KCl)	75.0	200	2.67
Sodium Chloride (NaCl)	58.0	8000	138
Sodium Carbonate (Na_2CO_3)	106	6360	60
Sodium Bicarbonate ($NaHCO_3$)	84.0	3360	40

The final exact pH was adjusted to 10.05 by addition of diluted aqueous HCl solution and measuring the pH on a pH meter (WTW pH 3310 with a SenTix® 41 electrode).

Method B

To a 1.5 mL-vial (screw top) was added the photochromic material **1** as powder and 250 µL of water. After complete dissolution, the solution was irradiated for 10 min at 365 nm. Subsequently, 250 µL of a 2× stock solution of sodium alginate in water was added and mixed by repetitive pipetting. Then, the mixture was irradiated at 455 nm for 10 min. Successfully formed gels were prepared in triplicates.

Melting temperatures were determined in triplicates. Gels were prepared as previously described. The vials were then mounted upside-down in a slowly stirred water bath (60 rpm) on a magnetic hotplate stirrer equipped with a thermometer. The water bath was heated (1.5 °C/min) until the gel started to melt and formed a sol.

*Table S 4: Hydrogelation experiments with alginate – **method B**.*

Composition of the solution (x mg + 500 µL H_2O)		Approx. Concentration		Description	T_m °C
Gelator **1**	Alginate	Gelator **1**	Alginate		
3	3	0.6 wt%	0.6 wt%	opaque, orange gel	89
2	2	0.4 wt%	0.4 wt%	opaque, orange gel	85
**1.5**	**1.5**	**0.3 wt%**	**0.3 wt%**	**clear, yellow gel**	**80**

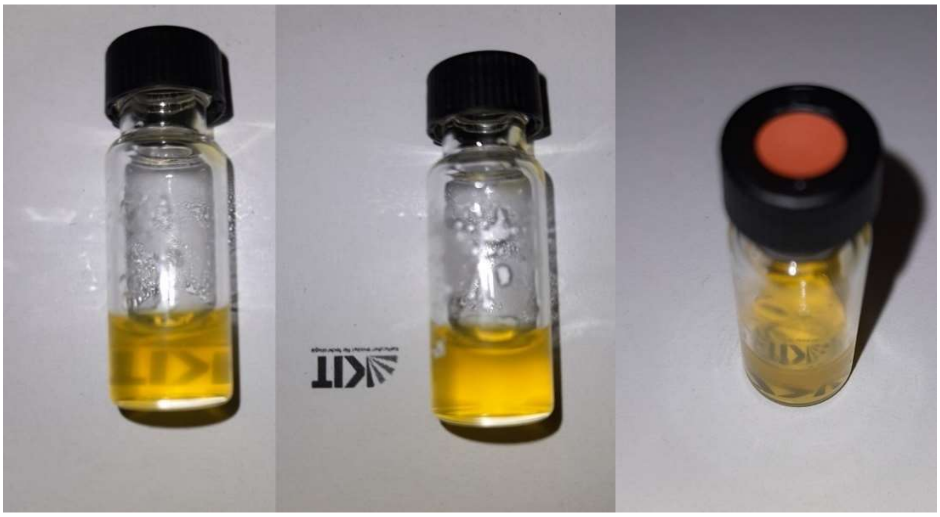

*Figure S 6: Pictures of a hydrogel formed by 0.3 wt% gelator **1** and 0.3 wt% alginate (Method B).*

5. Light induced gel-to-sol transition

According to the procedure **Method A** described in the previous section, gels were prepared with 0.6 wt% PAP-DKP-Lys_2 **1** and 0.6 wt% alginate in water. After equilibrating overnight, the samples were irradiated with 2 LEDs at 365 nm (10 W) for 15 min. Subsequently, the gels were inverted and one gel was irradiated at 455 nm for 15 min (A), while a second gel was kept in the dark (B, see **Figure S 7**). Sample A solidified at the vial top, while sample B was a highly viscous liquid.

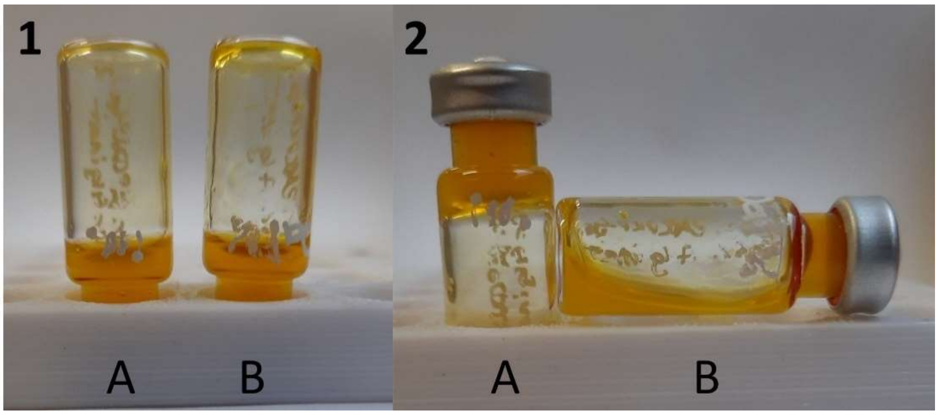

Figure S 7: 1) Gels after irradiation at 365 nm. 2) Gel A was irradiated at 455 nm, Gel B was kept in the dark.

6. Light-induced rhodamine release

Here we wanted to investigate how efficient are hydrogels based on the gelator **1** and sodium alginate in releasing encapsulated guest molecules by means of diffusion (in darkness) or dissipation of the inner gel structure upon irradiation with UV light. We have chosen the composition of 0.3 wt% of the gelator **1** and 0.3 wt% of alginate in 500 µL of water prepared by **Method B**. As described in section 4 of this supporting information, it forms a stable gel after irradiation of the mixed components at 455 nm. By this preparation method heat is avoided, which could have damaging impact on some cargo. Preparation with cargo was done as follows:

In a 1.5 mL glass vial (screw top) we mixed the photochromic gelator **1** (1.5 mg, powder) and water (245 µL). This solution was irradiated at 365 nm (10 min), then a 100× stock solution (5 µL) of the chosen cargo rhodamine dissolved in EtOH was added. Subsequently, a 2× stock solution of alginate (250 µl) was added, thoroughly mixed and the final mixture was irradiated at 455 nm for 10 min to obtain the final gel. Before a release experiment, the hydrogels were kept overnight in darkness at room temperature. Concentration of the cargo rhodamine was adapted to the HPLC detection range. 250 µg total mass of rhodamine were incorporated into the gels.

Quantification of the passive diffusion – cargo "leaking" from hydrogels in darkness:

500 µL of PBS buffer pH 7.4 was slowly added on top of a gel sample (on the wall of the vial) and immediately removed with a micropipette to wash away unbound or loosely bound guest molecules from the surface. Addition of fresh 500 µL of PBS buffer followed. The gel was incubated together with the buffer on the top in darkness. 500 µL of the liquid was collected after 5 min by gently turning the vial sideways and pipetting off the liquid from the side wall of the vial. Then, fresh 500 µL of PBS buffer was added on the side wall of the vial, incubated in darkness and removed after 5 min in the same way as described above. That process was repeated for the total duration of 40 min by collecting 9 subsequent volume aliquots. After that time, the gel remained visually unaffected.

Procedure of the light induced release:

To measure the release process upon UV light irradiation, we exactly repeated the procedure described above, but after initial washing of the gel surface the sample was placed in an irradiation chamber and illuminated with two 10 W LEDs (365 nm, from the distance of 5 cm).

Short breaks in irradiation were taken for the replacement of 500 µL aliquot with fresh 500 µL of PBS buffer every 5 min, but the overall irradiation time was 40 min. The irradiation time was sufficient to fully convert the gel samples into sol. All aliquots were weighted before the HPLC measurement to calculate the released amount of the substance. The concentration of the aliquots was calculated by a previously measured calibration curve.

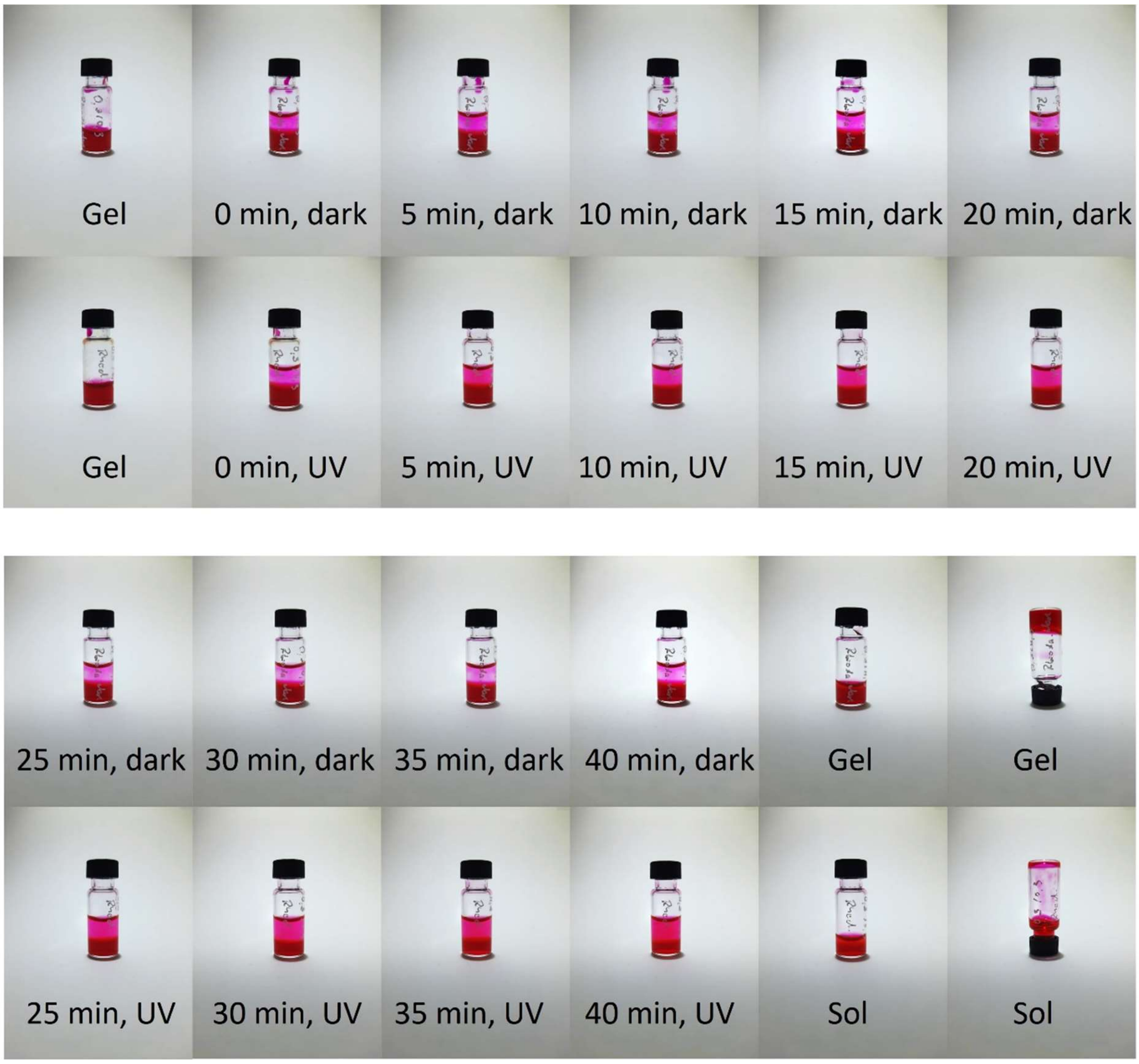

Figure S 8: Visual development of the gels and supernatant during the release experiment.

As a result, there was only minor leaking in the dark equilibrated gel (51.4 µg in total during 40 min), while there was a distinct release (112.5 µg in total during 40 min) from the irradiated gel, which can be seen by the color development and the quantified amount of released rhodamine (Figure S 8-Figure S 9 and Figure 11 in the manuscript).

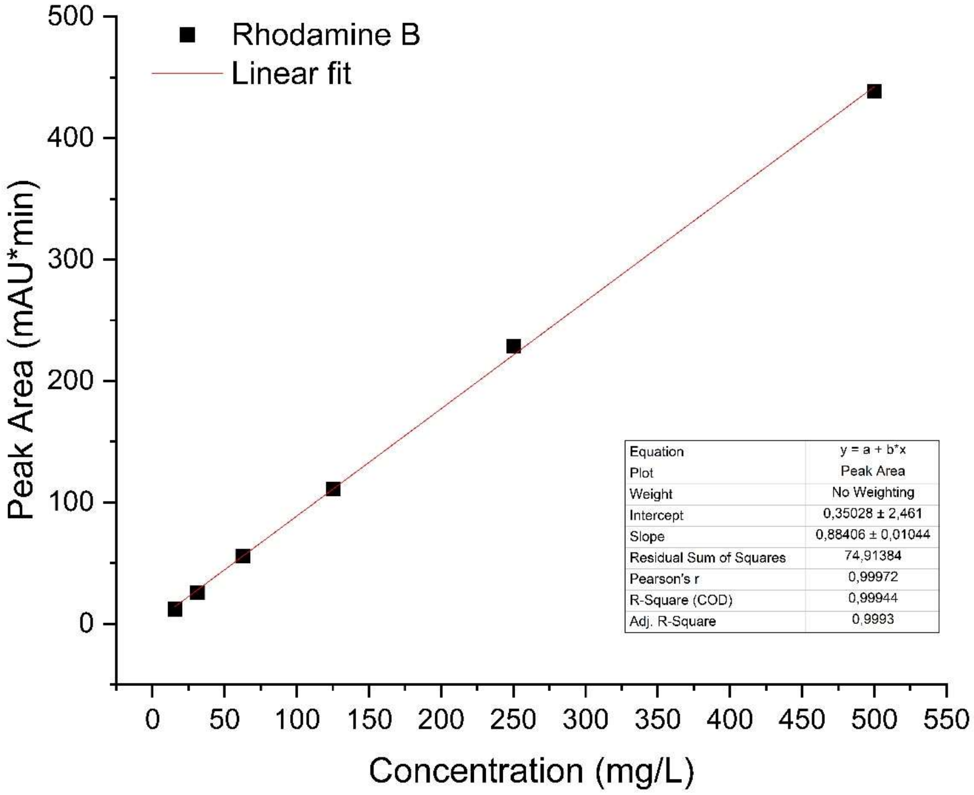

Figure S 9: The calibration curve for quantification of the rhodamine B release by HPLC

7. Diffusion of the gelator 1 after addition of Ca^{2+} ions

Sodium alginate rapidly forms gels upon addition of divalent ions, for example Ca^{2+}. Therefore, we have assessed the influence of an aqueous calcium salt solution on our composite gels made from alginate cross-linked with the gelator **1**. For this purpose, two gels composed of 0.6 wt% gelator **1** and 0.6 wt% alginate were prepared by **Method A** (see Figure S 10).

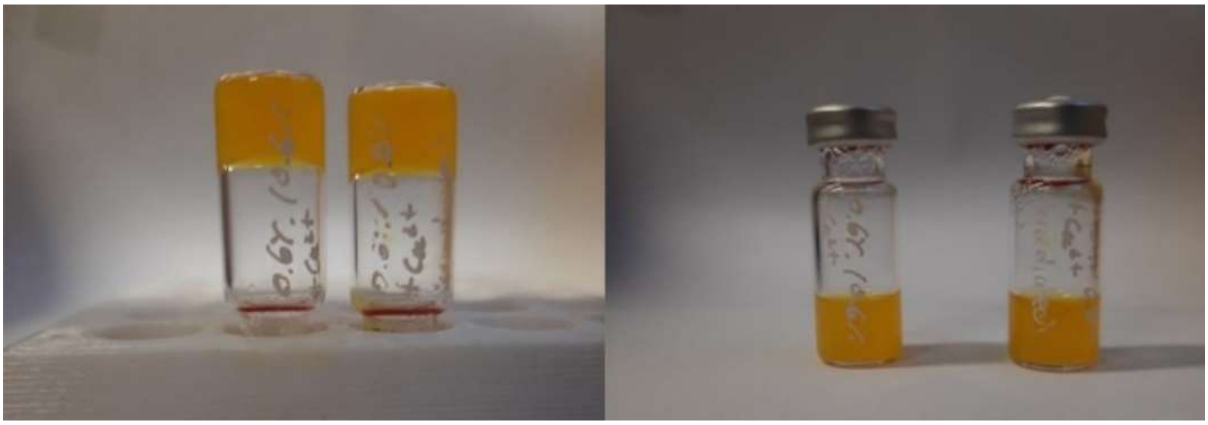

*Figure S 10: Hydrogels at 0.6 wt% gelator **1** and 0.6 wt% alginate prepared by Method A.*

On top of these gels was added 500 µL of a 10 wt% solution of $CaCl_2$ in water and equilibrated for 1 h.

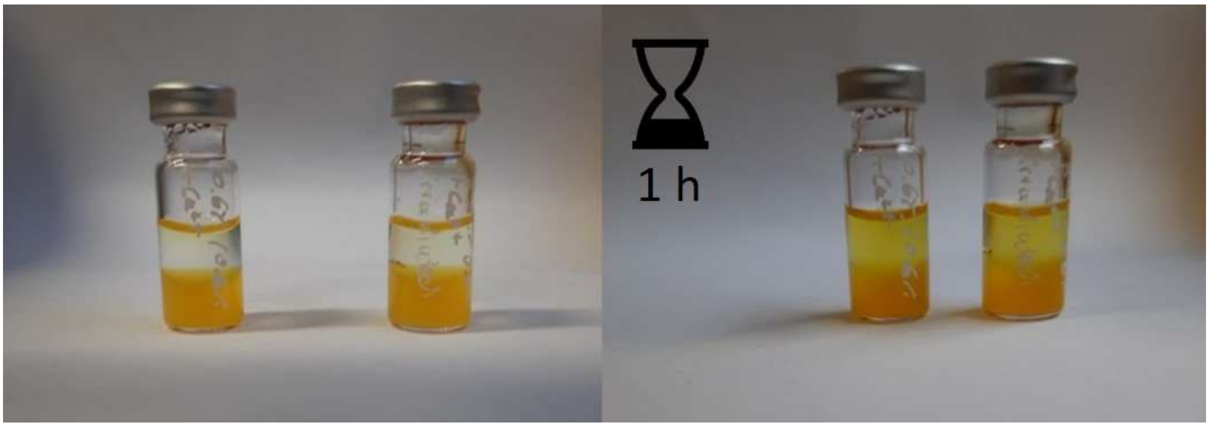

Figure S 11: 500 µL $CaCl_2$ solution (10 wt% in water) added on top of the gels for 1 Subsequently, the supernatant was removed and fresh $CaCl_2$ solution was added. Then, one vial was kept in the dark, while the second one was irradiated for 1 h (365 nm). Next, the supernatant was removed, and the procedure was repeated four times in total. The supernatant of the irradiated vials was colorful, while the dark vials were less colored (Figure S 12).

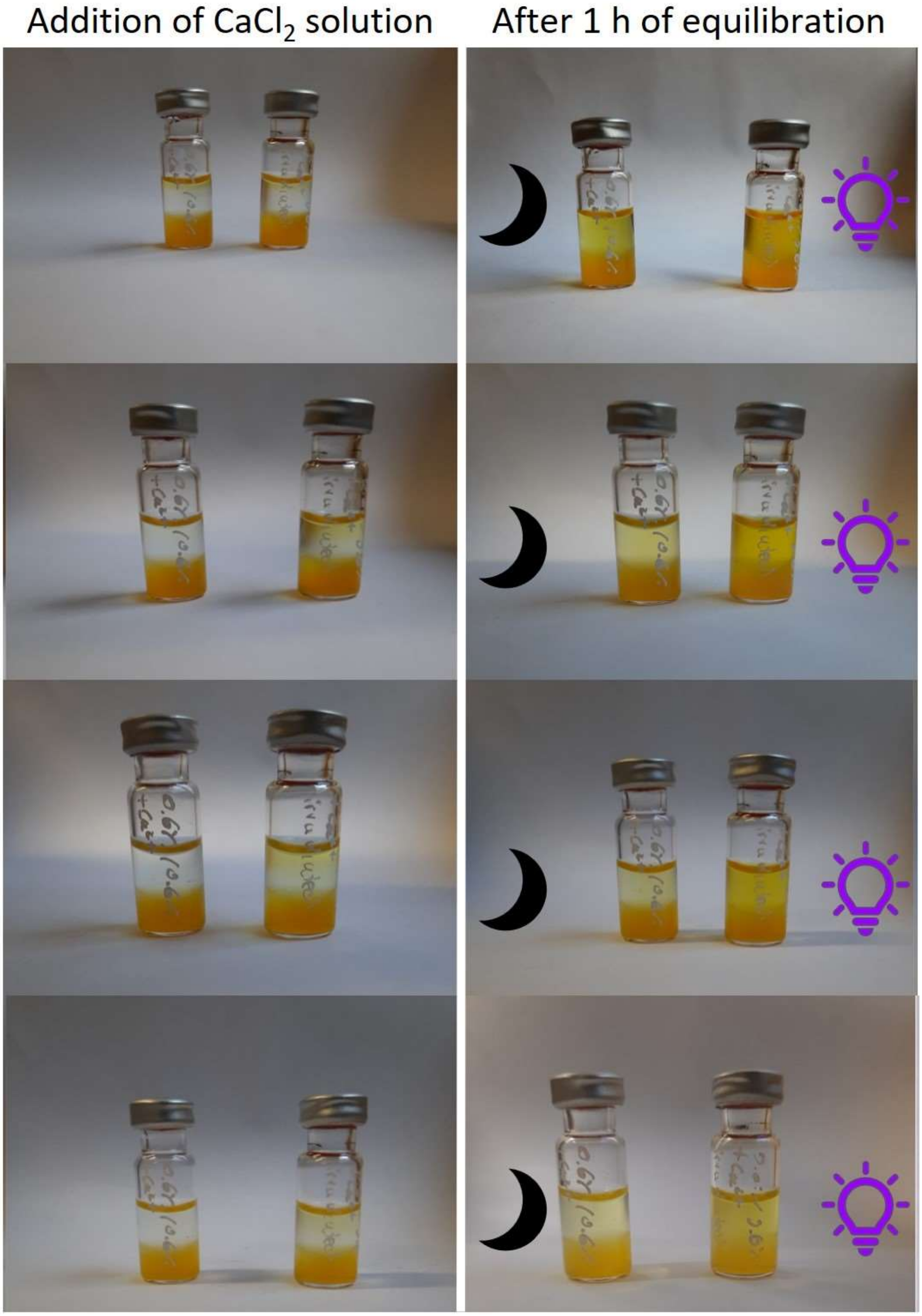

Figure S 12: Four irradiation / dark cycles. Left row: fresh $CaCl_2$ solution was added to the top. Right row: 1 h equilibration either at 365 nm or in the dark.

After four cycles, in both irradiated and dark equilibrated vial, a stable gel remained. The gel in the irradiated vial was less opaque and less colorful compared to the non-irradiated vial (Figure S 13).

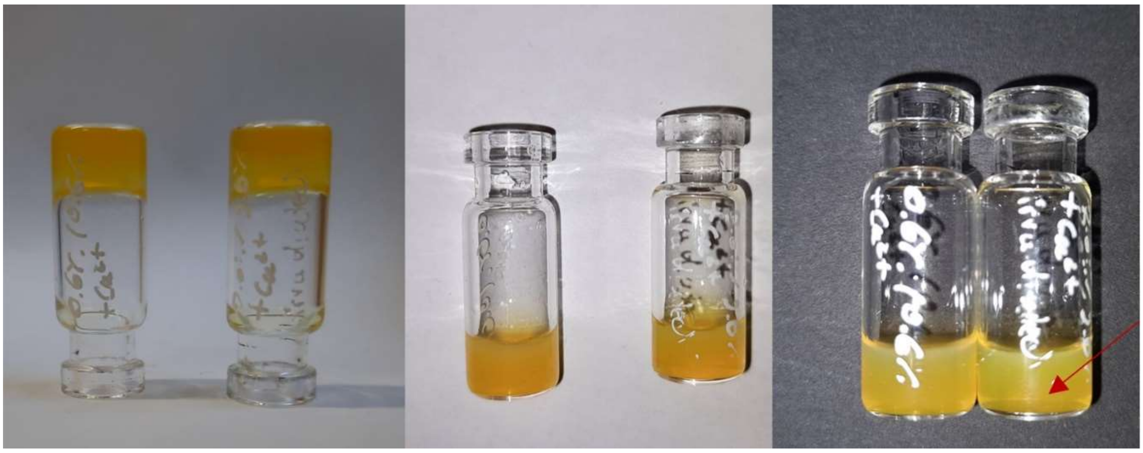

Figure S 13: Remaining gel after the Ca^{2+} experiment. In each picture the right vial was irradiated. The gel in the irradiated vial is less opaque, the bottom plate of the vial is clearly visible (red arrow).

This experiment demonstrated that our gelator **1** / alginate composite gels can be transformed to Ca^{2+} / alginate gels and our gelator **1** is subsequently removable by irradiation.

8. Cell viability assays of the hydrogelator 1

Hela cells were grown in DMEM (Dulbecco`s Modified Eagle Medium) which was modified with 10% FCS (fetal calf serum) and 1% penicillin/streptomycin solution (10,000 units/mL of penicillin and 10,000 µg/mL of streptomycin) in a humid incubator at 37 °C with 5% CO_2. Cells were detached from the surfaces with Trypsin-EDTA (0.25%) from Gibco®. Cells were washed with PBS (Phosphate-Buffered Saline) from Gibco®.

96-well plates (Table 1) with a flat bottom were prepared by filling all wells on the outer border with 200 µL PBS and the remaining wells with 100 µL of a cell suspension (30.000 cells/mL) in DMEM. The prepared plate was incubated overnight to ensure cell attachment to the well-bottom and cell growth.

Table 1: Scheme of the 96-well plate. The sample positions were filled as follows: row 2 with the positive control (all cells are dead), row 11 with the negative control (all cells are alive) and row 3 to 10 with one specific concentration respectively.

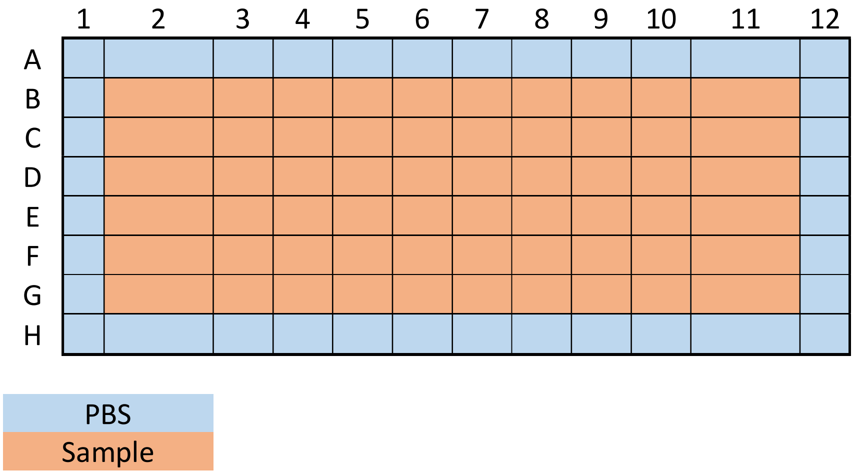

For the dilution-series of each compound, a stock solution of DMEM modified with 0.25% DMSO was prepared to ensure that all cells are treated with the same conditions. Consequently, the first sample of the dilution series was prepared by dissolving the substance in DMSO and adding a specific amount of this solution to a specific amount of non-modified DMEM so that a final concentration of 0.25% DMSO is reached.

To apply the substances to the 96-well plate, the DMEM was removed without disturbing the cells grown in the plate and adding subsequently 100 µL to each well. To ensure the same treatment to the control rows, the DMEM was removed from the wells and DMSO-modified DMEM (100 µL) was added to the corresponding wells. The 96-well plate was incubated for 48 h.

The positive control was treated with 5 µL of Triton™ X-100 detergent (10% solution (w/v)) per well for at least 5 min before adding 15 µL of MTT dye-solution (Cell Proliferation Kit I (MTT) from Roche) to all sample wells and incubating for 3 h in the dark. 100 µL of stop solution (Cell Proliferation Kit I (MTT) from Roche) was added after incubation to stop the reduction of MTT to

formazan, thus preventing overreaction and enabling solubilization of formazan crystals. After 24 h of solubilization in the incubator, the plate was read out with a plate reader (BioTek® EPOCH^2, Gen5 Data Analysis) by measuring the absorption of each well at 595 nm.

The raw data was processed as followed by first subtracting the positive control (all cells are dead) from all measured values in one row to remove background absorption. Each concentration was measured sixfold per plate therefore (Triplicates; 18 values in total), the values for each concentration and the negative control (all cells alive) were averaged and the standard deviation was calculated. The cell viability was calculated as a percentage of the negative control and normalized by assuming the highest obtained viability as 100%.

Table S 5: Results of the cell viability assay.

PAP-(DKP-Lys)$_2$ **365 nm irradiated**				PAP-(DKP-Lys)$_2$ **dark adapted**			
Conc.	Conc. [M]	Cell viability [%]	Stdev	Conc.	Conc. [M]	Cell viability [%]	Stdev
1 mM	1,00E-03	84	11	1 mM	1,00E-03	51	15
100 µM	1,00E-04	100	11	100 µM	1,00E-04	100	10
10 µM	1,00E-05	95	11	10 µM	1,00E-05	99	15
1 µM	1,00E-06	94	13	1 µM	1,00E-06	96	12
100 nM	1,00E-07	96	12	100 nM	1,00E-07	97	12
10 nM	1,00E-08	96	10	10 nM	1,00E-08	94	11
1 nM	1,00E-09	96	11	1 nM	1,00E-09	90	10
100 pM	1,00E-10	90	10	100 pM	1,00E-10	88	8

9. Microscopy images

Transmission Electron Microscopy (TEM):

For the Transmission Electron Microscopy images, two 1.5 wt% hydrogel samples of **1** in PBS buffer and two mixed samples (0.6 wt% **1** and 0.6 wt% alginate) in H_2O were prepared as described in the Gelation experiments 4. After cooling, the samples were equilibrated overnight at room temperature. Of each composition one sample was irradiated at 365 nm (10 W LED) until liquefaction. The resulting samples were added as small droplet to carbon-coated copper grids (400 mesh). The supernatant was removed carefully with a lint-free sheet and the grid was dried under atmospheric pressure. Examination was carried out on a Philips CM200 FEG transmission electron microscope, operated at 200 kV accelerating voltage. All images were recorded defocused.

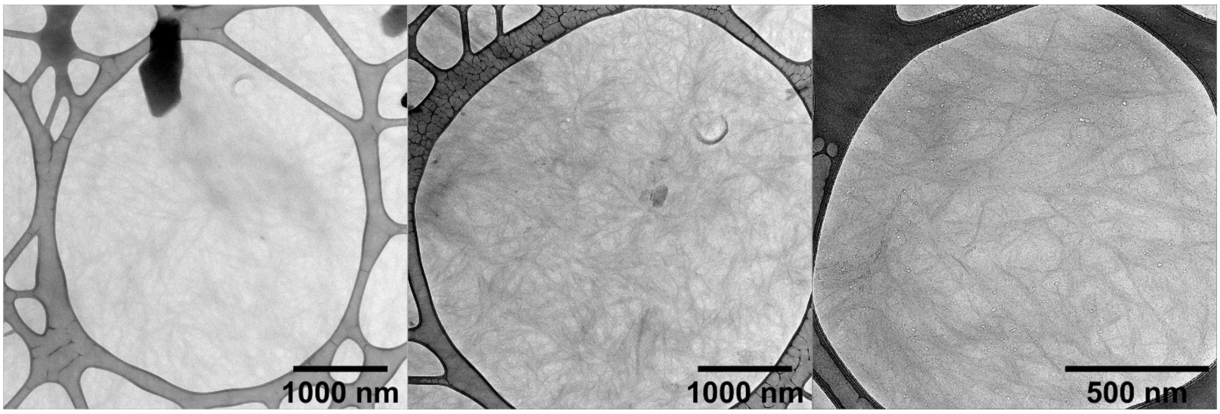

*Figure S 14: TEM images of Hydrogelator **1** 1.5 wt% in PBS buffer; dark adapted.*

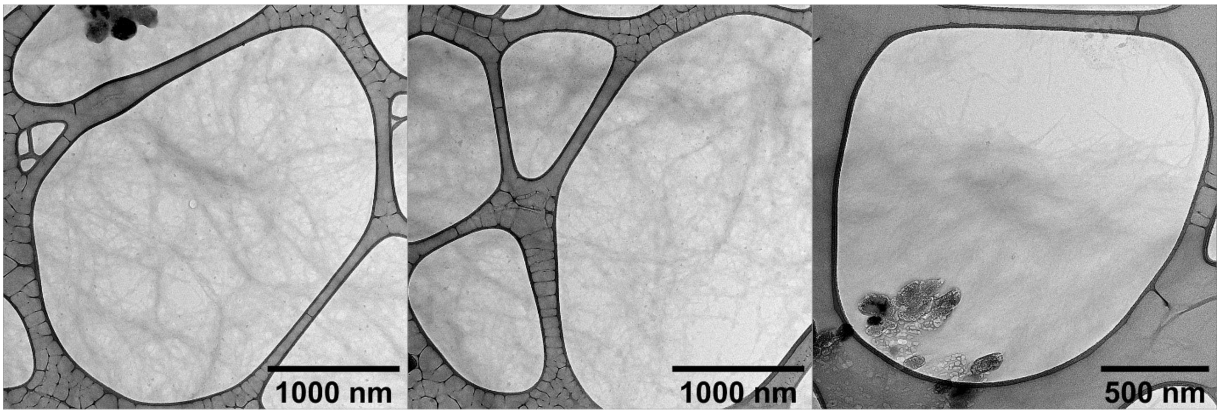

*Figure S 15: TEM images of Hydrogelator **1** 1.5 wt% in PBS buffer; irradiated.*

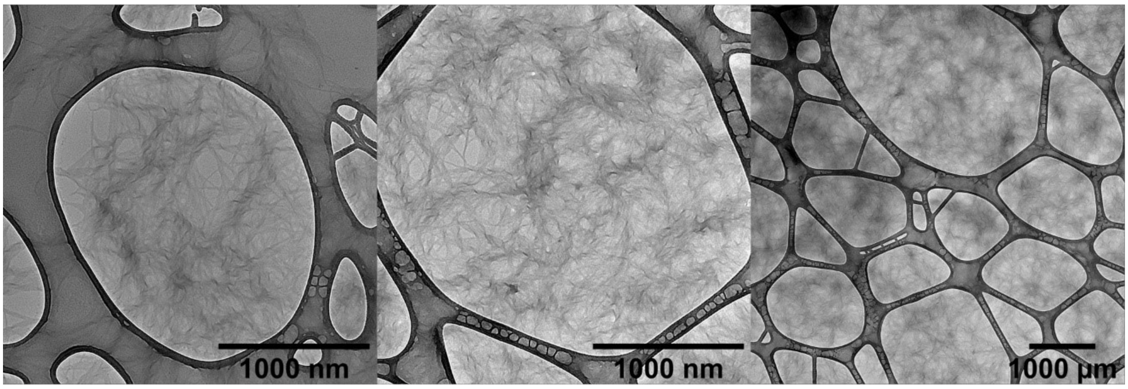

*Figure S 16: TEM images of 0.6 wt% Hydrogelator **1** and 0.6 wt% alginate in H_2O; dark adapted.*

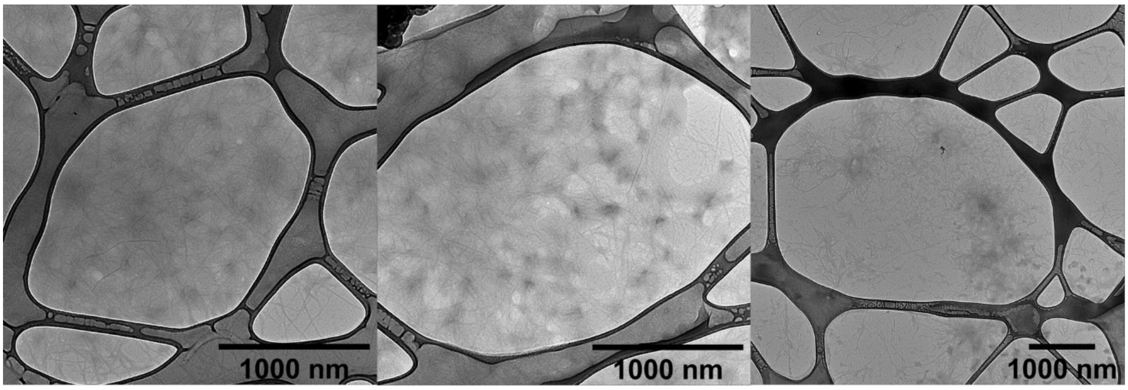

*Figure S 17: TEM images of 0.6 wt% Hydrogelator **1** and 0.6 wt% alginate in H_2O; dark adapted.*

Scanning Electron Microscopy (SEM):

For the Scanning Electron Microscopy images, a sample with 1.5 wt% hydrogelator **1** was prepared with PBS buffer and a sample with 0.6 wt% hydrogelator **1** and 0.6 wt% alginate was prepared with diH_2O. The resulting material was freeze dried by lyophilization and then coated with a thin layer of platinum.

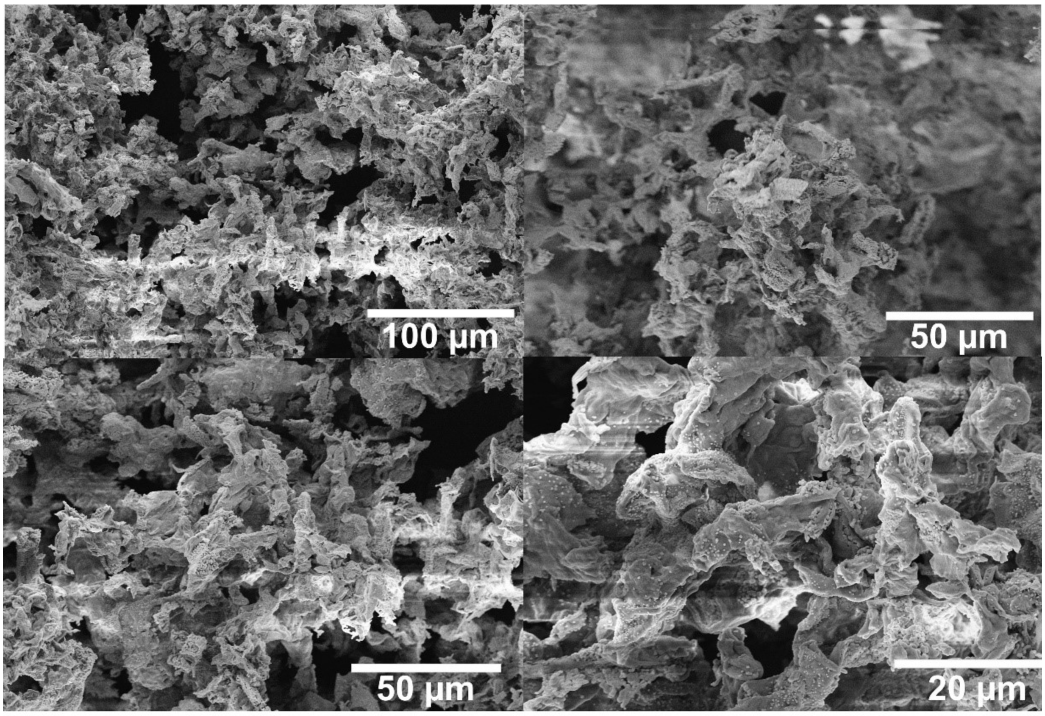

*Figure S 18: SEM images of 1.5 wt% Hydrogelator **1** in PBS buffer.*

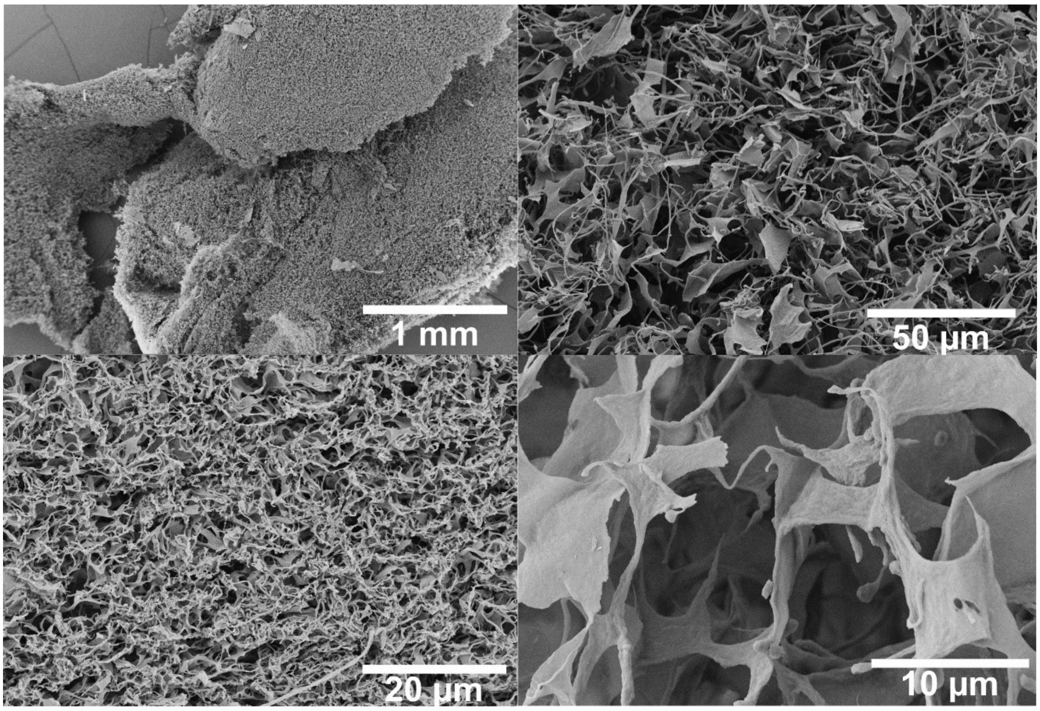

*Figure S 19: SEM images of 0.6 wt% Hydrogelator **1** and 0.6 wt% alginate in H_2O.*

10. Rheological experiments

Oscillatory shear experiments were conducted on a strain-controlled ARES-G2 (TA Instruments) rheometer with a parallel plate geometry (25 mm diameter and 1 mm gap). A volume of 0.5 mL of a sample consisting of 0.6 wt% hydrogelator **1** and 0.6 wt% alginate (prepared as described in 4. Gelation experiments) was poured onto the lower plate. The Temperature was maintained at 20 °C by a Peltier element. A strain sweep was conducted by varying the strain amplitude in the range γ = 0.01 – 37 % at an angular frequency ω = 6.28 rad s^{-1} to determine the linear viscoelastic (LVE) regime. A frequency sweep was recorded in the range ω = 0.19 - 452 rad s^{-1} at γ = 0.5 % within the LVE regime.

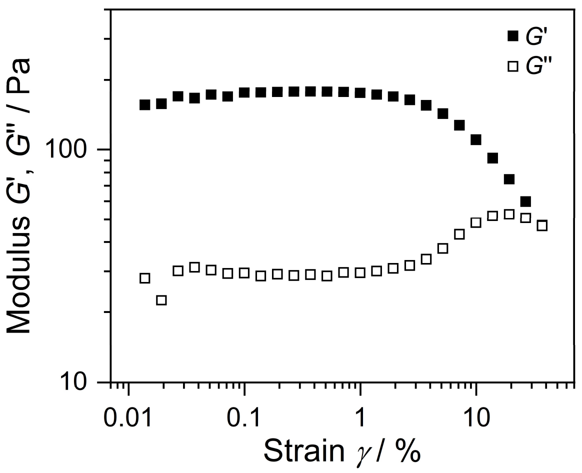

*Figure S 20 Strain sweep of a sample consisting of 0.6 wt% hydrogelator **1** and 0.6 wt% alginate at an angular frequency ω = 6.28 rad s^{-1}.*

11. NMR spectra of the synthesized compounds

L-Phe-(4-NO2)-OH, (*S*)-4-Nitrophenylalanine (2)

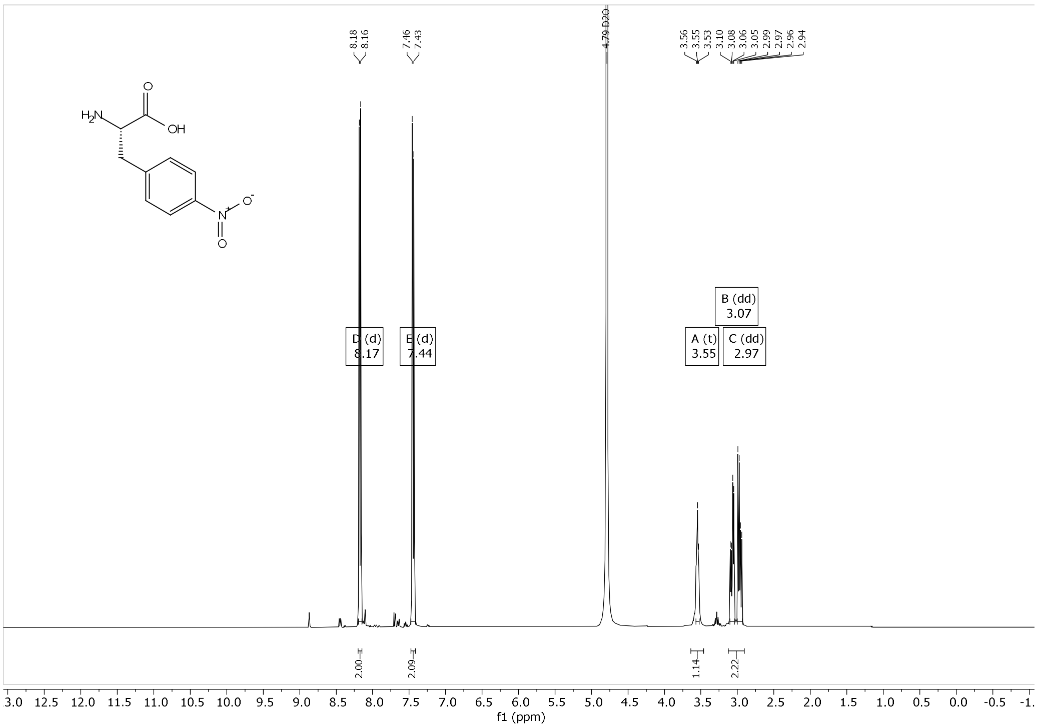

*Figure S 21: ^{1}H-NMR-spectrum (400 MHz, D_2O with KOH) of the compound **2**.*

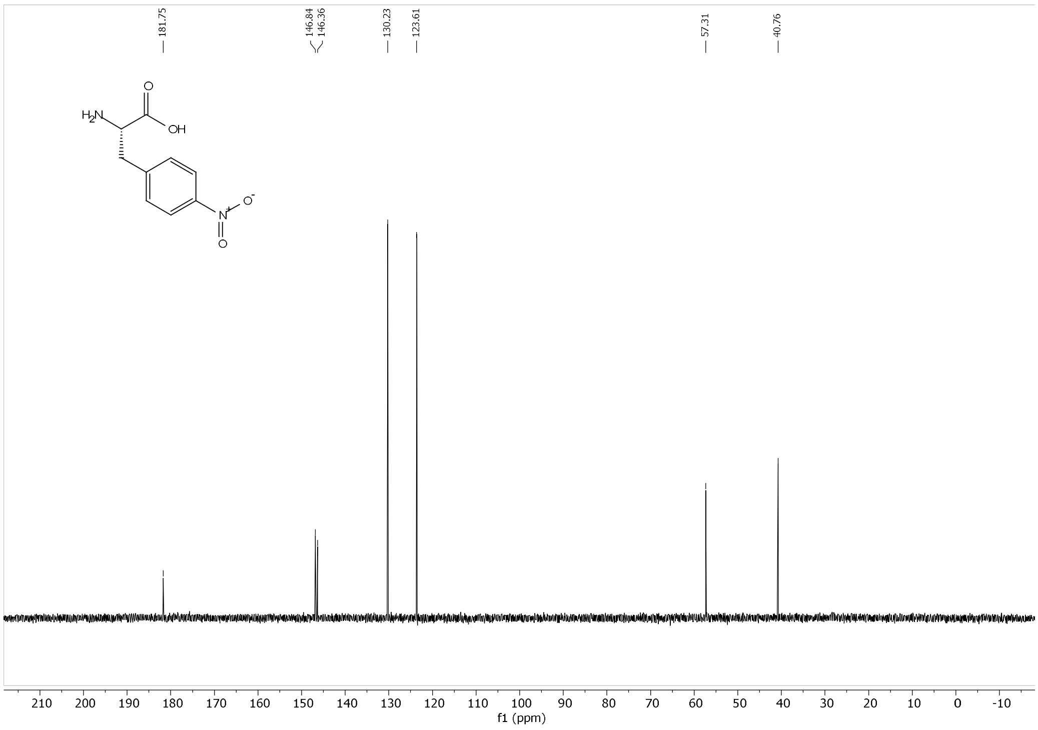

*Figure S 22: ^{13}C-NMR-spectrum (101 MHz, D_2O with KOH) of the compound **2**.*

L-Phe-(4-NO$_2$)-OMe · HCl, (*S*)-4-Nitrophenylalanine methyl ester hydrochloride (3)

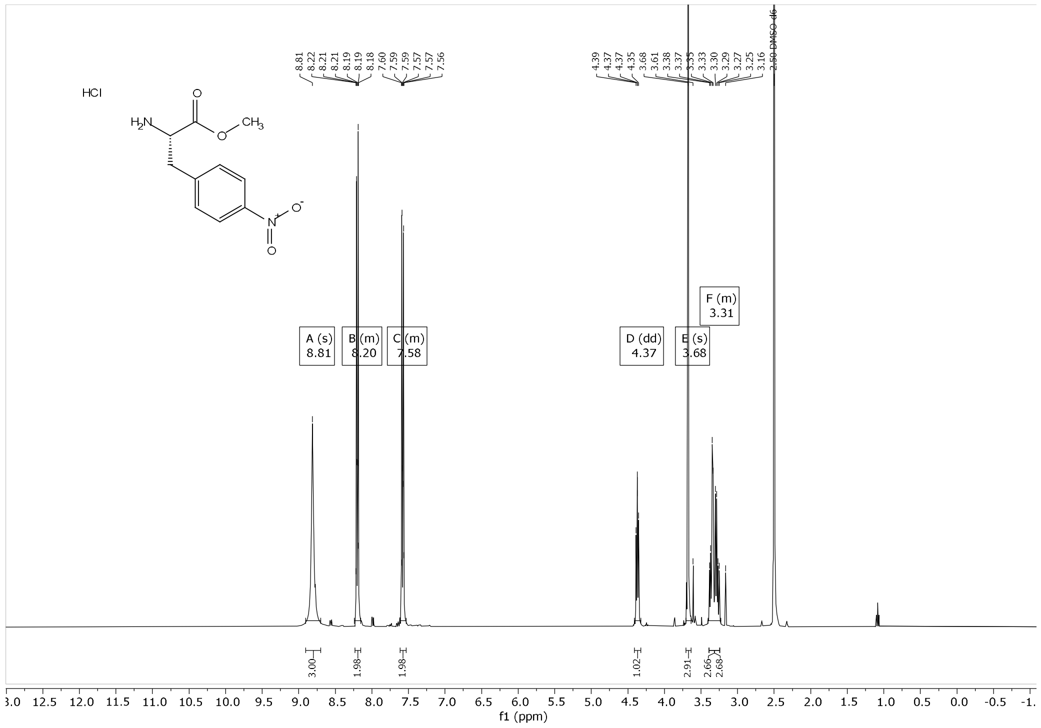

*Figure S 23: ^{1}H-NMR-spectrum (400 MHz, DMSO-d_6) of the compound **3**.*

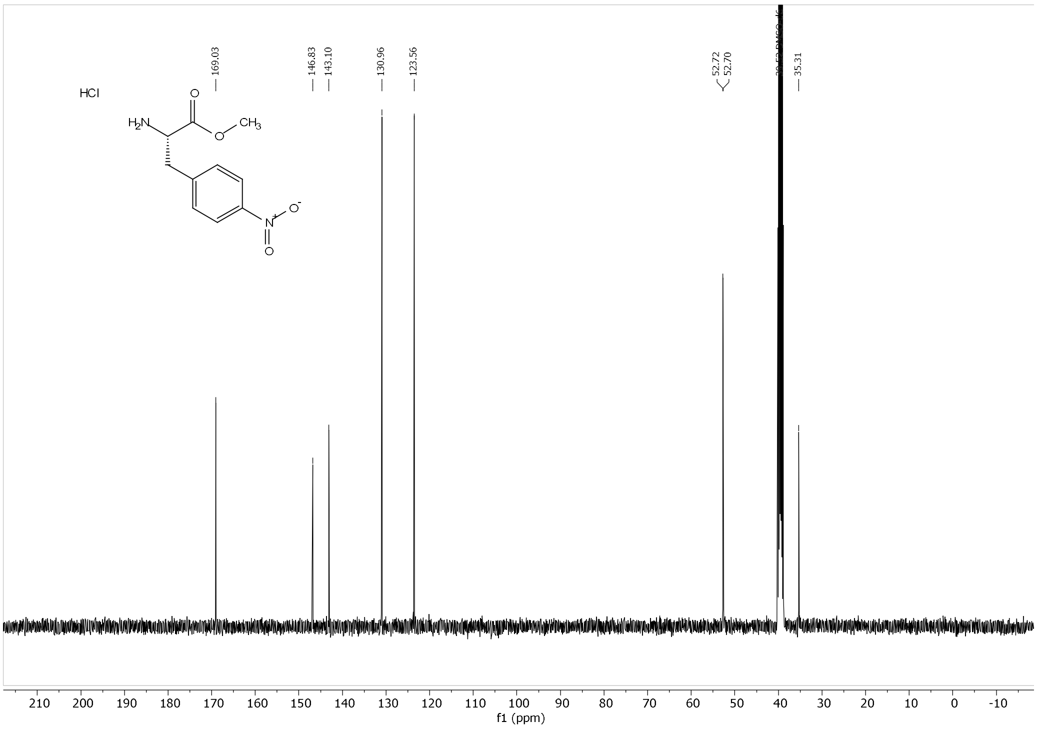

*Figure S 24: ^{13}C-NMR-spectrum (101 MHz, DMSO-d_6) of the compound **3**.*

Boc-L-Phe-(4-NO$_2$)-OMe, Methyl (*S*)-2-((*tert*-butoxycarbonyl)amino)-3-(4-nitrophenyl)propanoate (4)

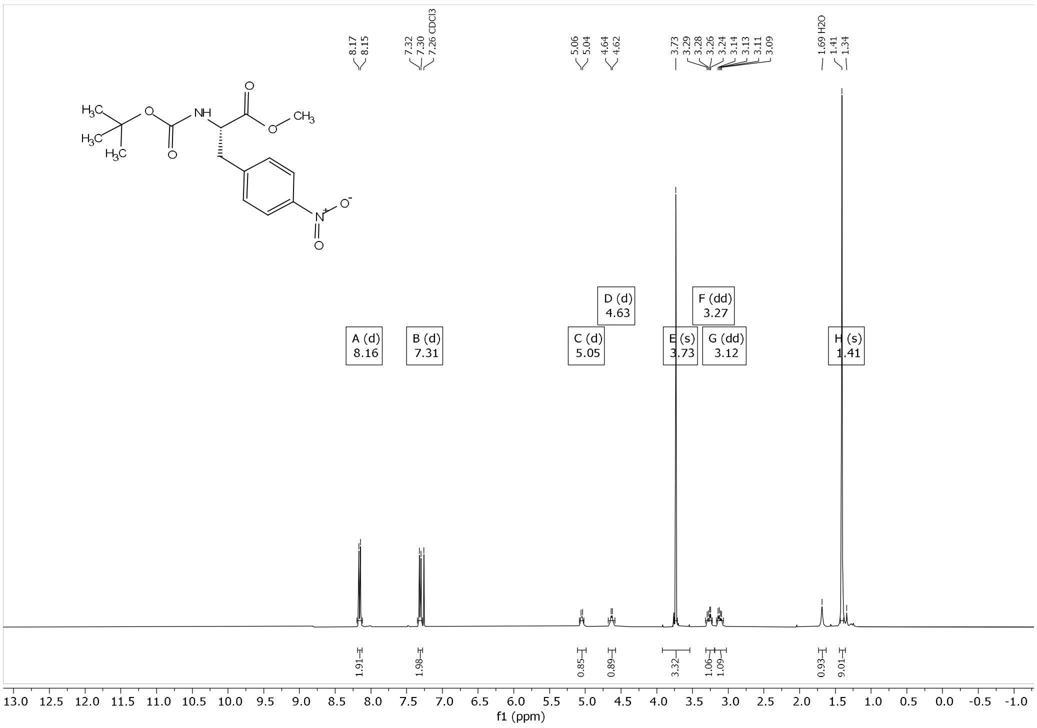

*Figure S 25: ^{1}H-NMR-spectrum (400 MHz, $CDCl_3$) of the compound **4**.*

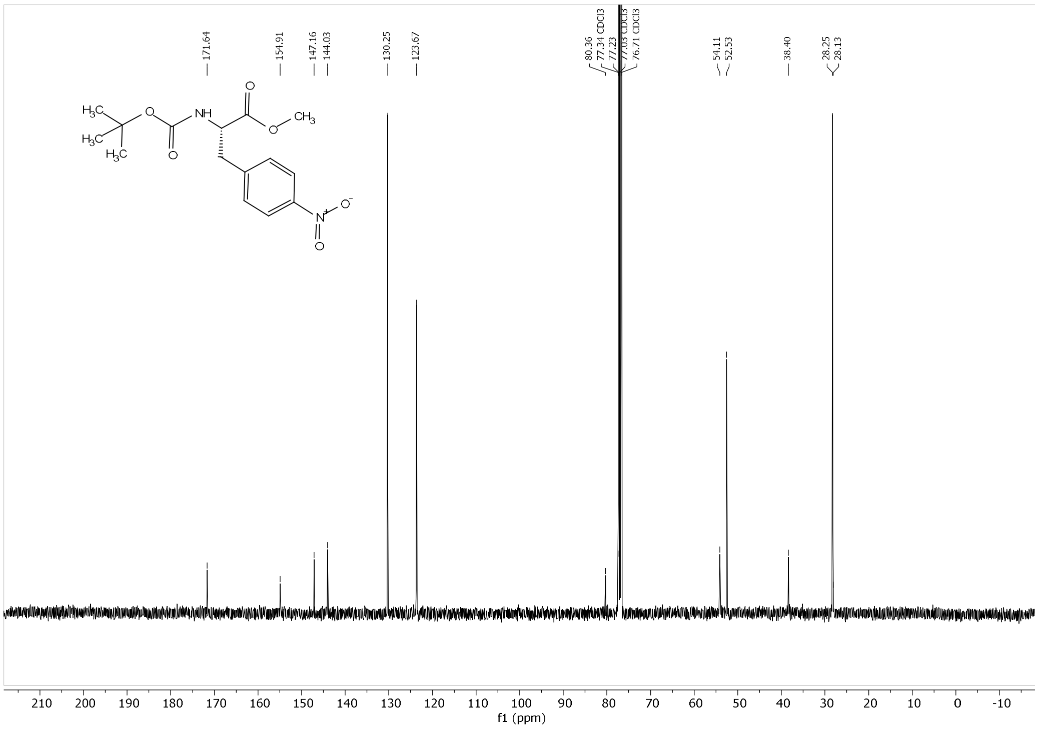

Figure S 26: ^{13}C-NMR-spectrum (101 MHz, $CDCl_3$) of the compound ***4****.*

Boc-L-Phe-(4-NH$_2$)-OMe, Methyl (*S*)-3-(4-aminophenyl)-2-((tert-butoxycarbonyl)amino)propanoate (5)

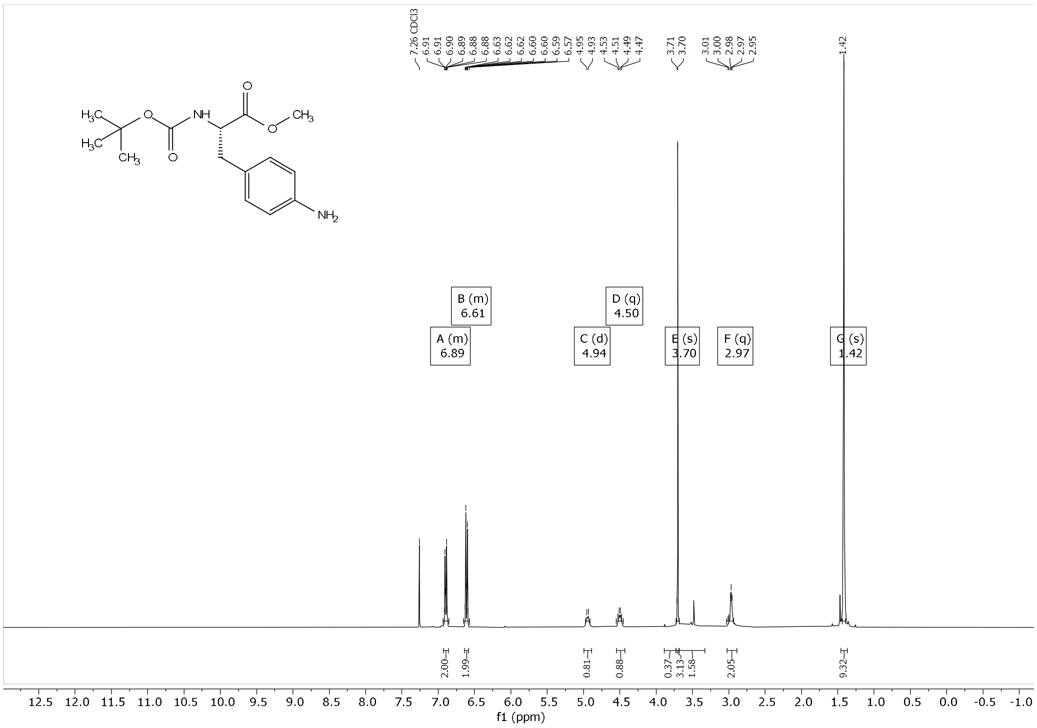

*Figure S 27: ^{1}H-NMR-spectrum (400 MHz, $CDCl_3$) of the compound **5**.*

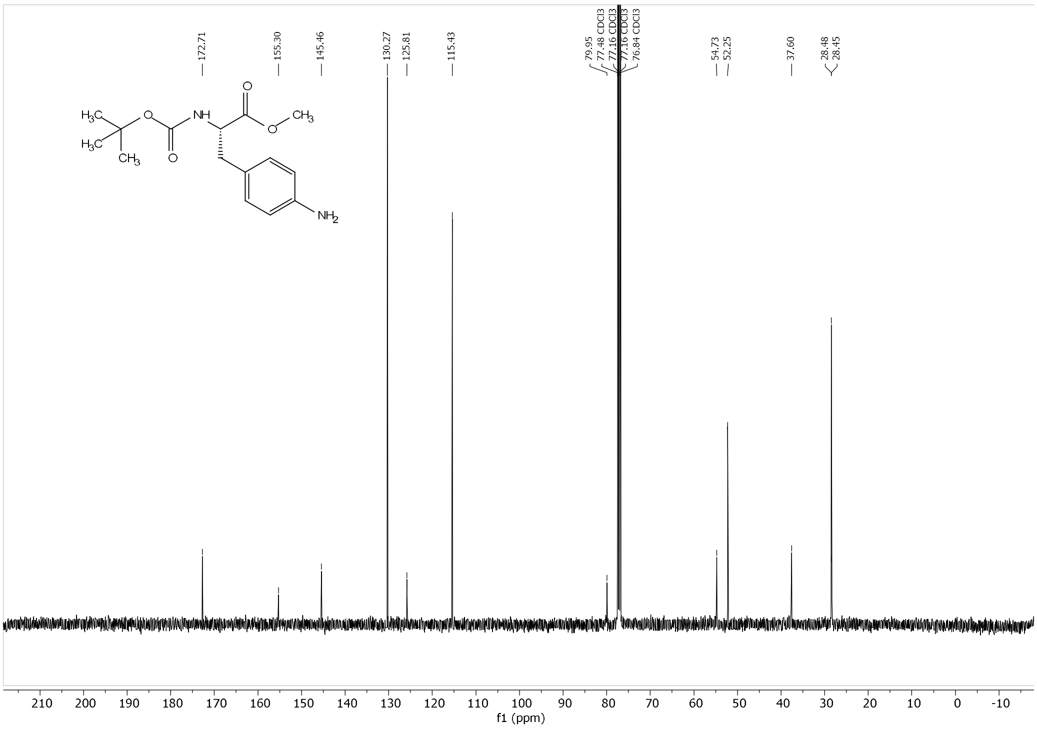

*Figure S 28: ^{13}C-NMR-spectrum (101 MHz, $CDCl_3$) of the compound **5**.*

(*S*)-sym-(Boc)$_2$-PAP-OMe, Dimethyl 3,3'-(((*E*)-diazene-1,2-diyl)bis(4,1-phenylene))(2*S*,2'*S*)-bis(2-((tert-butoxycarbonyl)amino)propanoate) (6)

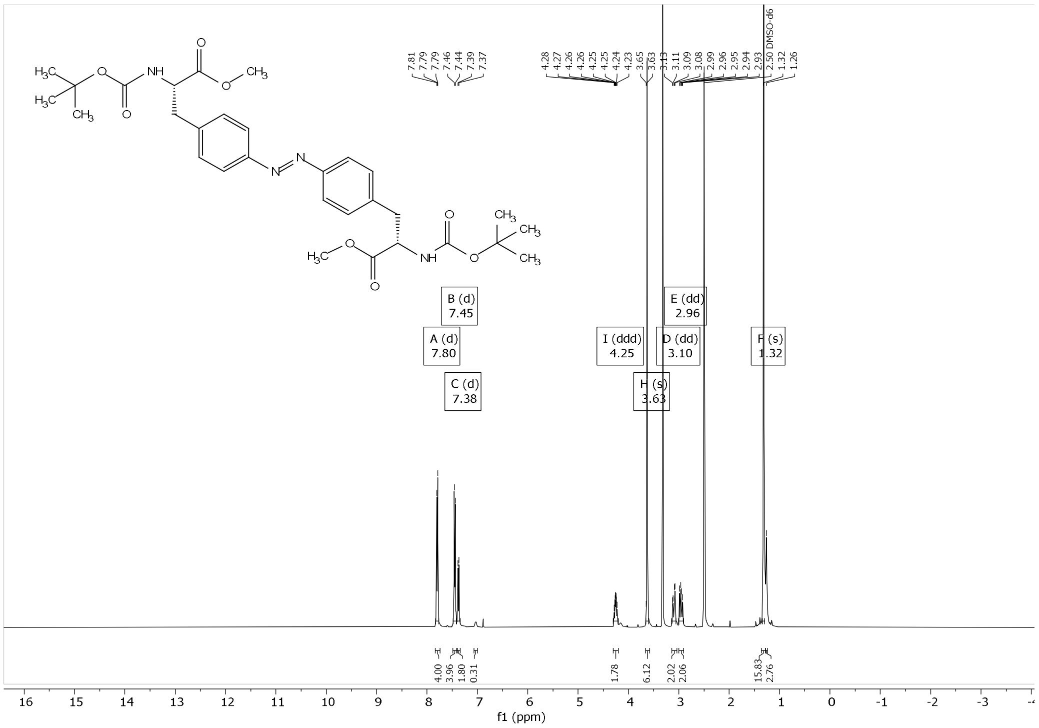

*Figure S 29: ^{1}H-NMR-spectrum (400 MHz, DMSO-d$_6$) of the compound **6**.*

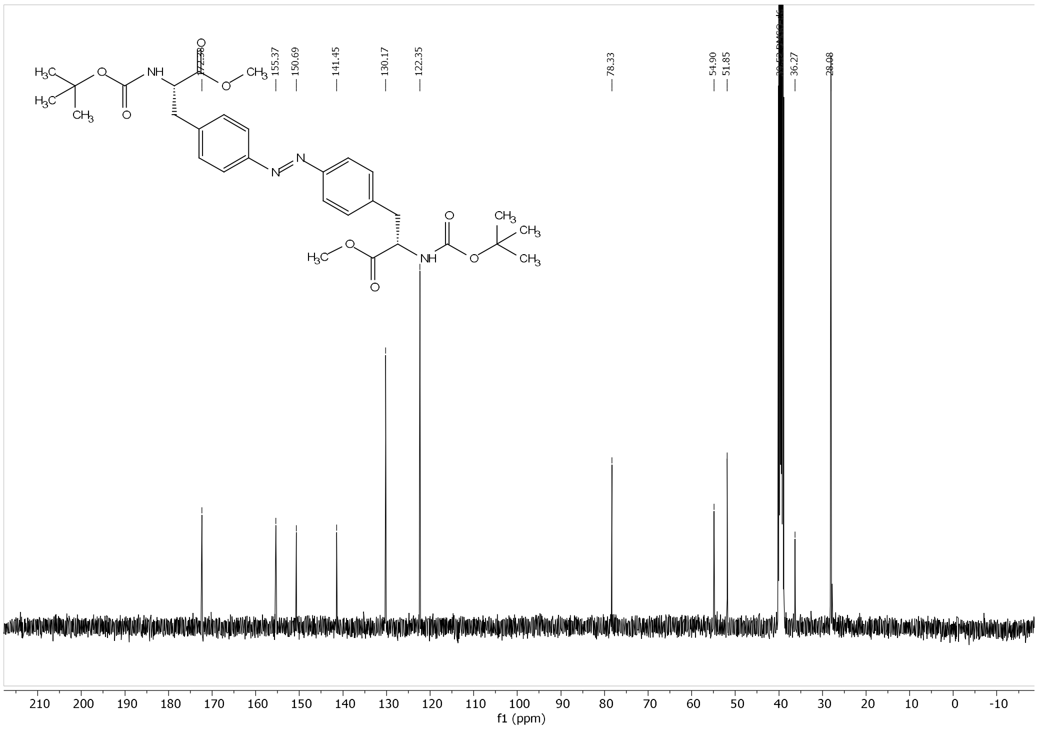

*Figure S 30: ^{13}C-NMR-spectrum (101 MHz, DMSO-d$_6$) of the compound **6**.*

(*S*)-sym-(Boc)$_2$-PAP-OH, (2*S*,2'*S*)-3,3'-(((*E*)-diazene-1,2-diyl)bis(4,1-phenylene))bis(2-((tert-butoxycarbonyl)amino)propanoic acid) (7)

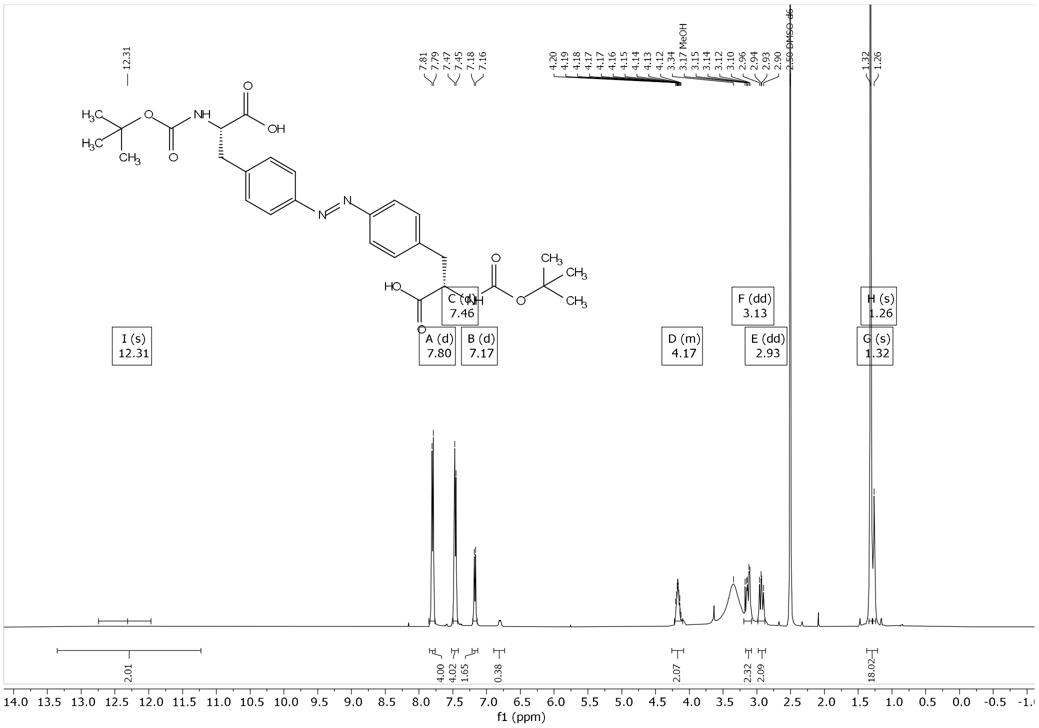

*Figure S 31: ^{1}H-NMR-spectrum (400 MHz, DMSO-d$_6$) of the compound **7**.*

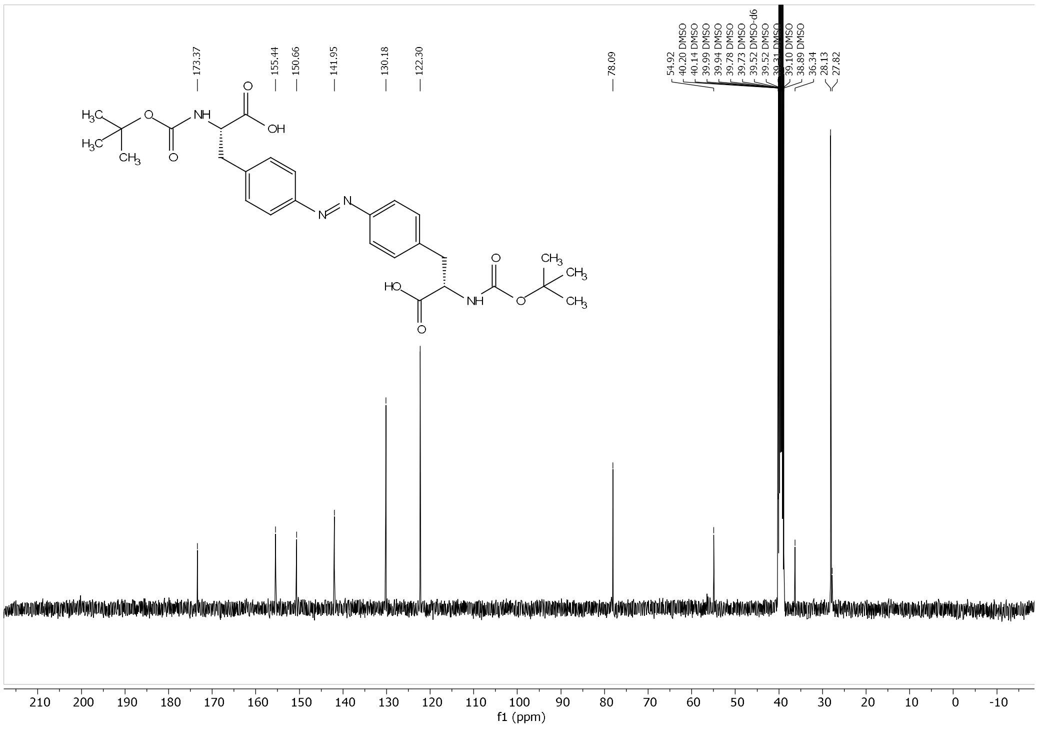

*Figure S 32: ^{13}C-NMR-spectrum (101 MHz, DMSO-d$_6$) of the compound **7**.*

sym-(Boc)Lys$_2$-(Boc)PAP-OMe, Dimethyl 2,2'-(((2*S*,2'*S*)-3,3'-(((*E*)-diazene-1,2-diyl)bis(4,1-phenylene))bis(2-((tert-butoxycarbonyl)amino)propanoyl))bis(azanediyl))(2*S*,2'*S*)-bis(6-((tert-butoxycarbonyl)amino)hexanoate) (8)

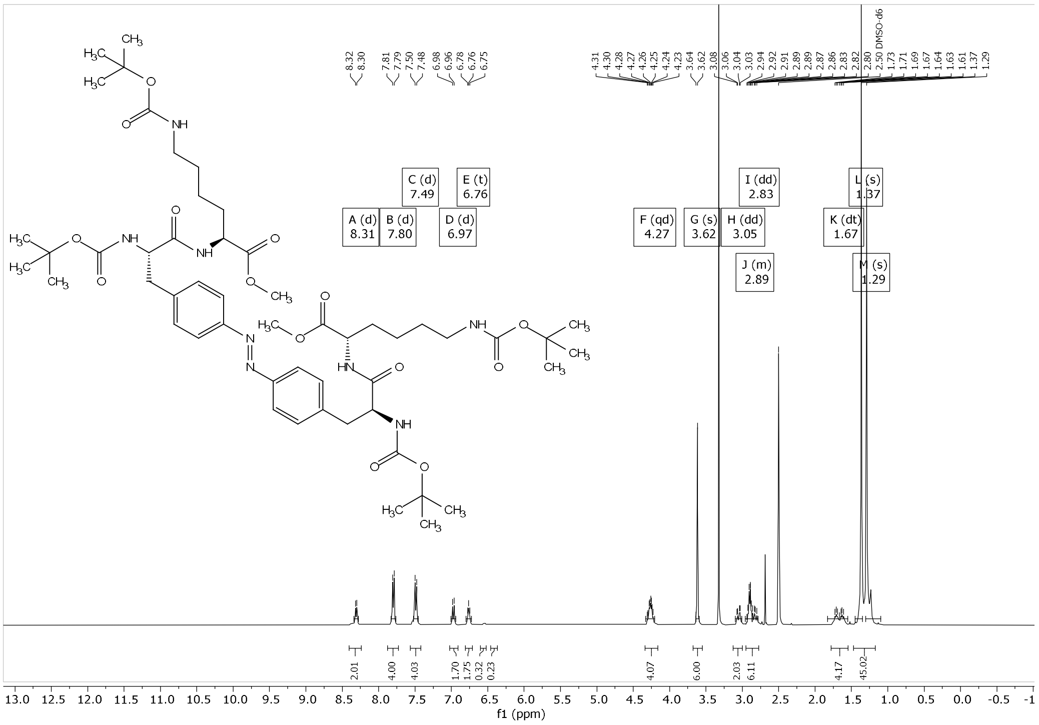

*Figure S 33: ^{1}H-NMR-spectrum (400 MHz, DMSO-d_6) of the compound **8**.*

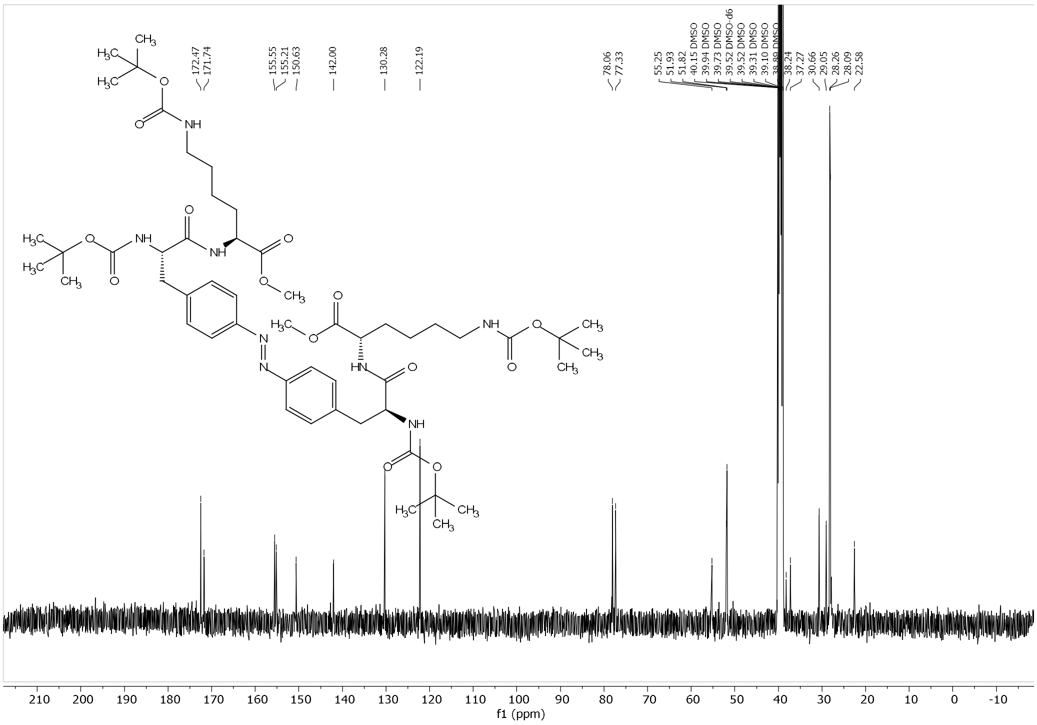

*Figure S 34: ^{13}C-NMR-spectrum (101 MHz, DMSO-d_6) of the compound **8**.*

Sym-PAP-Lys-OMe·TFA$_n$, Dimethyl 2,2'-(((2*S*,2'*S*)-3,3'-(((*E*)-diazene-1,2-diyl)bis(4,1-phenylene))bis(2-aminopropanoyl))bis(azanediyl))(2*S*,2'*S*)-bis(6-aminohexanoate) bis(trifluoroacetate) (9)

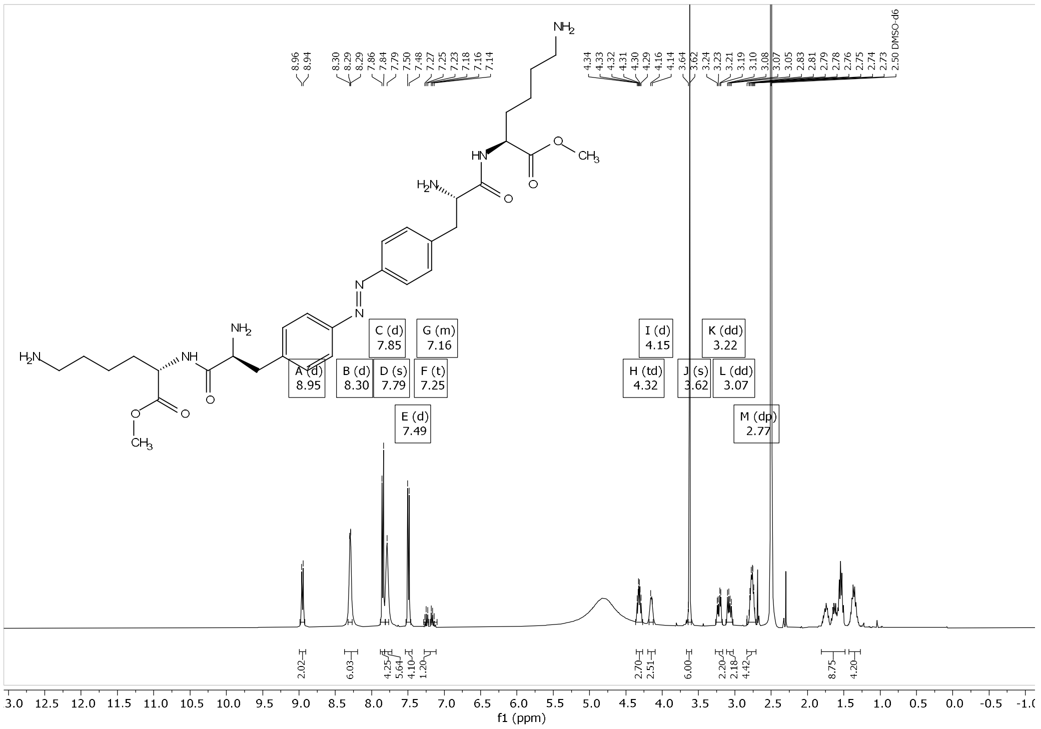

Figure S 35: ^{1}H-NMR-spectrum (400 MHz, DMSO-d$_6$) of the compound ***9****.*

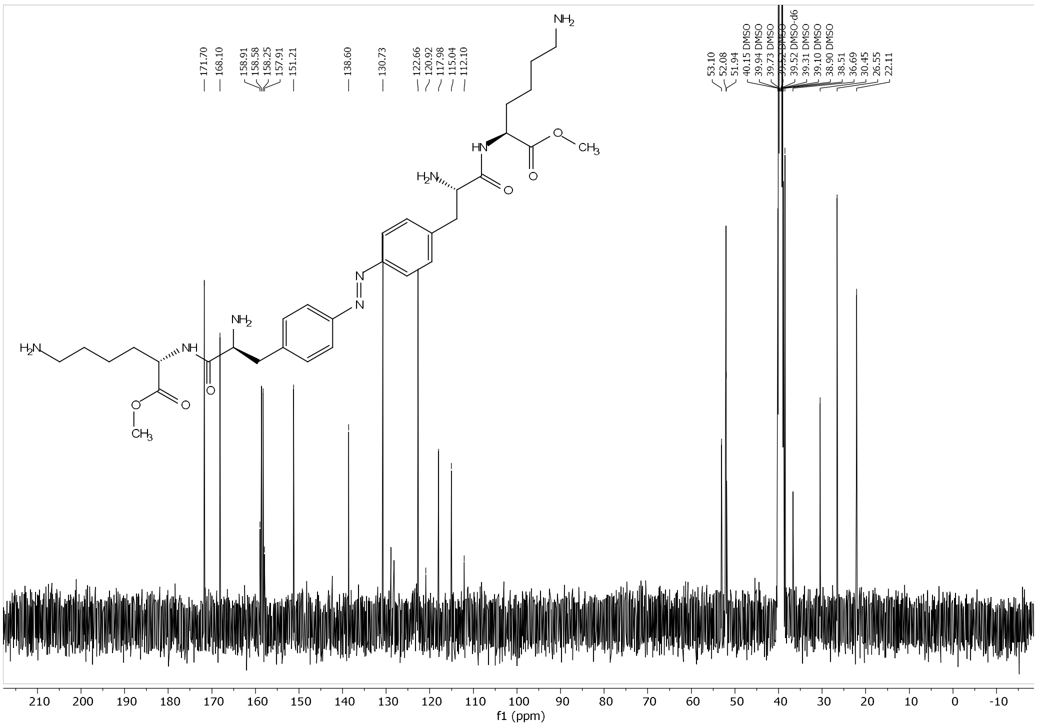

*Figure S 36: ^{13}C-NMR-spectrum (101 MHz, DMSO-d_6) of the compound **9**.*

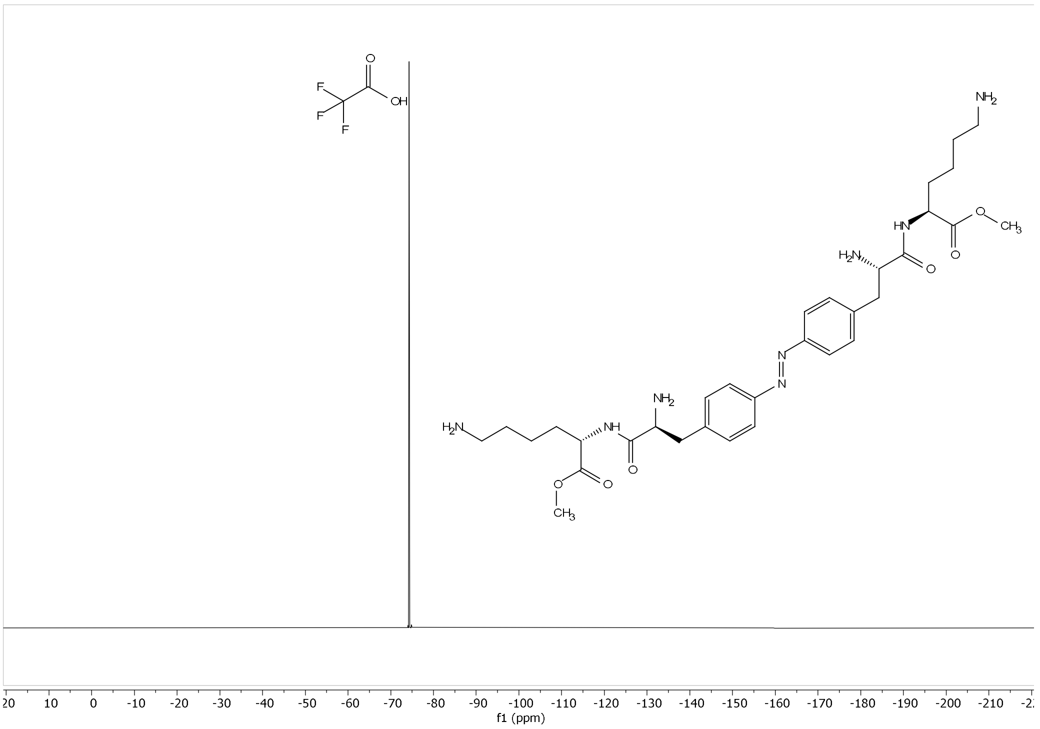

*Figure S 37: ^{19}F-NMR-spectrum (376 MHz, DMSO-d_6) of the compound **9**.*

PAP-DKP-Lys$_2$, (3*S*,3'*S*,6*S*,6'*S*)-6,6'-((((*E*)-diazene-1,2-diyl)bis(4,1-phenylene))bis(methylene))bis(3-(4-aminobutyl)piperazine-2,5-dione) bis(trifluoroacetate) (1)

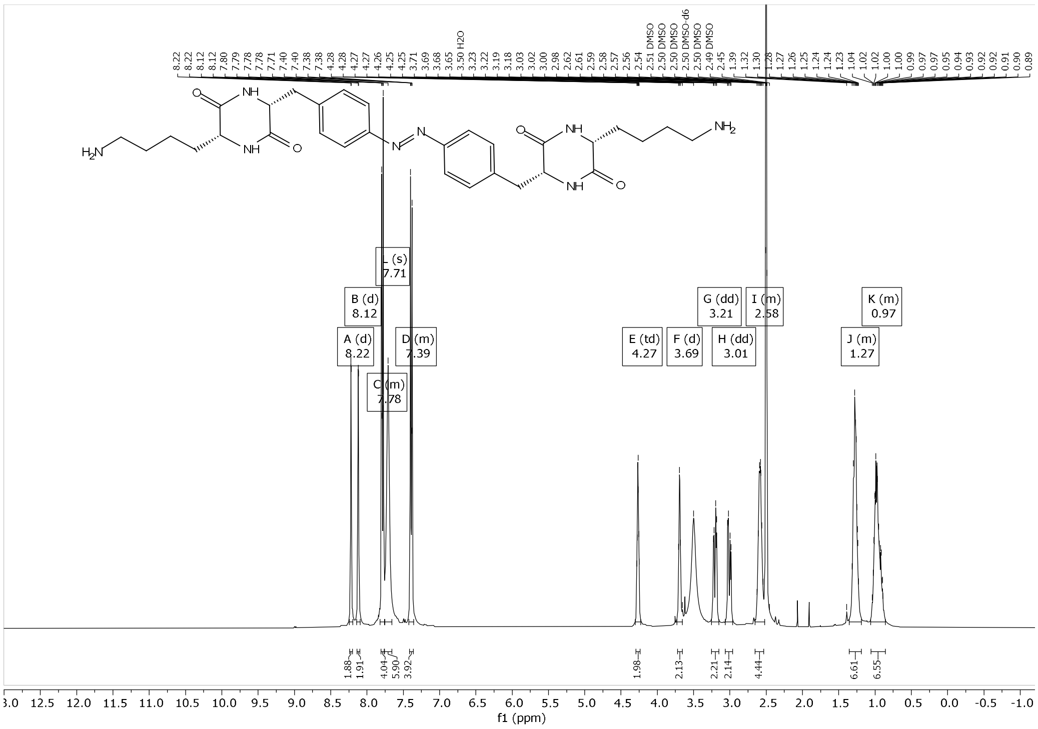

*Figure S 38: ^{1}H-NMR-spectrum (400 MHz, DMSO-d$_6$) of the compound **1**.*

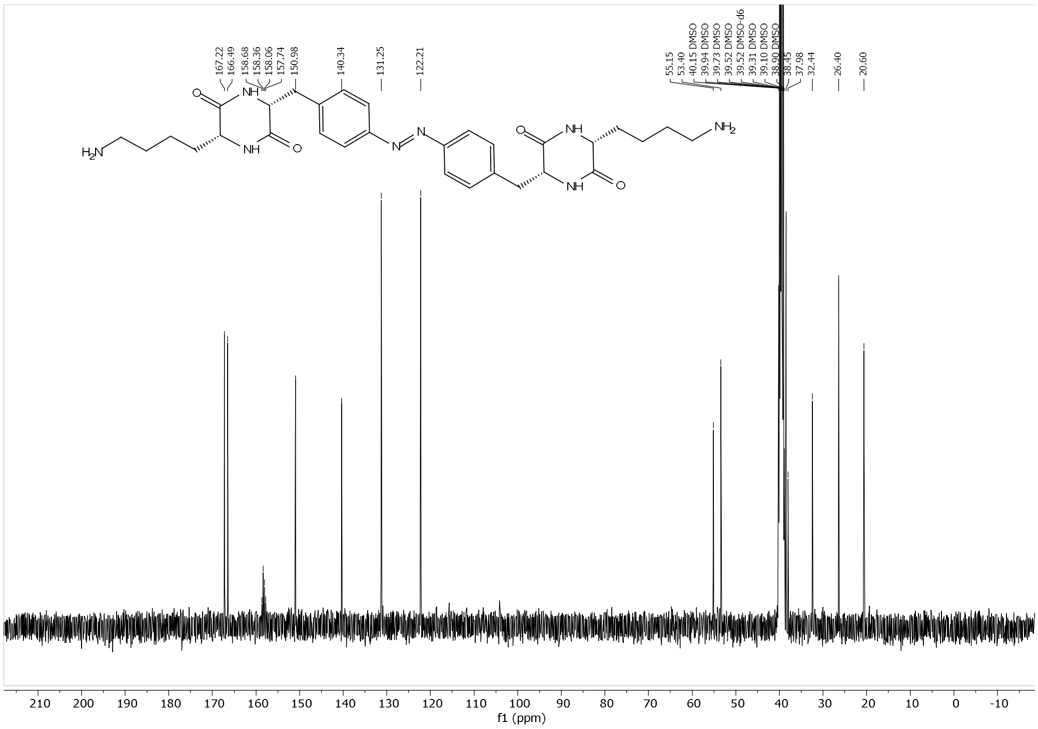

*Figure S 39: ^{13}C-NMR-spectrum (101 MHz, DMSO-d$_6$) of the compound **1**.*

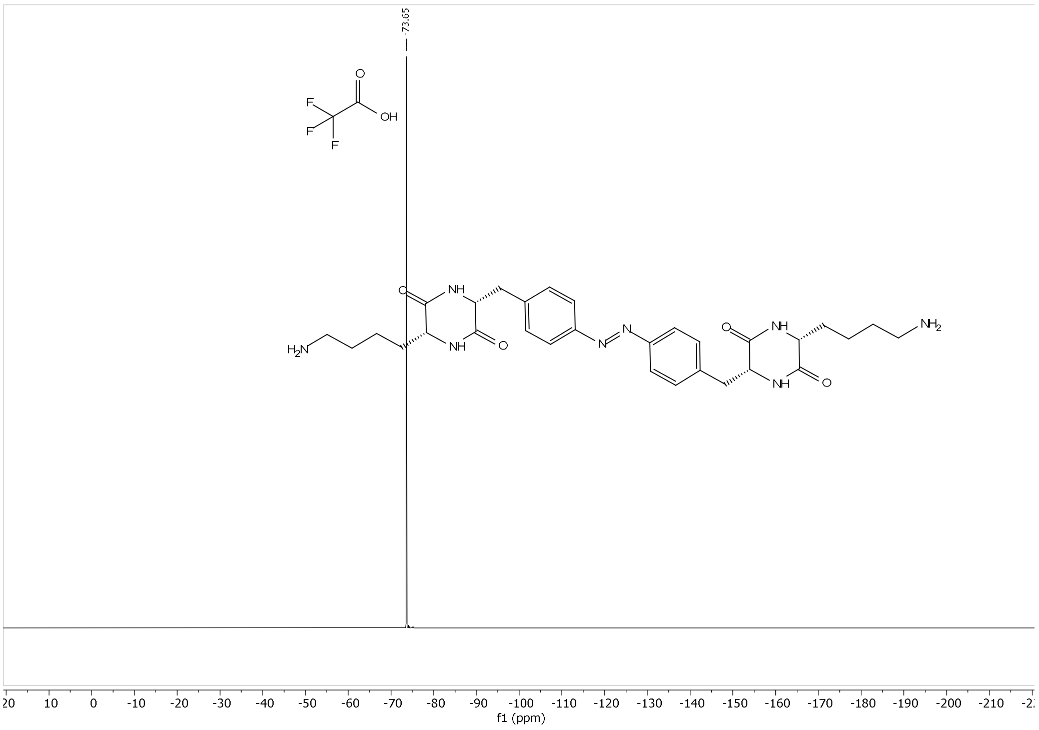

*Figure S 40: ^{19}F-NMR-spectrum (376 MHz, DMSO-d_6) of the compound **1**.*

Alfa Aesar

Certificate of analysis

Product No.:	A18565
Product:	Alginic acid sodium salt, very low viscosity
Lot No.:	10212201

Appearance:	Cream powder
Loss on Drying:	8.72% (105°C/constant weight)
pH:	6.0 (1% solution)
Viscosity:	7 mPa (1% solution)
Identification (FTIR):	Conforms

This document has been electronically generated and does not require a signature.

Order our products online **www.alfa.com**

ThermoFisher SCIENTIFIC

Figure S 41: Very low viscosity alginate – certificate of analysis

12. References

[1] H. E. Gottlieb, V. Kotlyar, A. Nudelman, *The Journal of Organic Chemistry* **1997**, *62*, 7512-7515.
